# Computer-Aided Drug Design for Type 2 Diabetes-Related Enzymes: Polarizable and Nonpolarizable Simulations of Enzyme-Inhibitor Complexes

**DOI:** 10.1021/acs.jcim.6c01734

**Published:** 2026-09-30

**Authors:** Esmat Mohammadi, Justin A. Lemkul

**Affiliations:** † Department of Chemical Engineering, 1757 Virginia Tech, Blacksburg, Virginia 24061, United States; ‡ Department of Biochemistry, Virginia Tech, Blacksburg, Virginia 24061, United States; § Center for Computer-Aided Drug Design, Virginia Tech, Blacksburg, Virginia 24061, United States

## Abstract

Carbohydrate-digesting enzymes, including pancreatic α-amylase and maltase-glucoamylase (MGAM), are important therapeutic targets for type 2 diabetes due to their role in regulating glucose production. In this study, molecular dynamics (MD) simulations were performed to investigate the stability and interaction patterns of inhibitors identified through SILCS-based virtual screening. Representative ligands for the N-terminal and C-terminal domains of MGAM (NtMGAM, CtMGAM), and α-amylase were parametrized using both the nonpolarizable CHARMM and polarizable Drude force fields based on quantum mechanical (QM) reference data. MD simulations of the apo enzymes show that their overall structures remain stable, with flexibility primarily localized to regions surrounding the catalytic pockets. Simulations of the enzyme–ligand complexes demonstrate that the ligands remain associated with the binding regions throughout the trajectories. Comparison between the two force fields reveals distinct interaction patterns: CHARMM interactions are largely driven by a small number of dominant contacts, whereas the Drude model captures a more extensive and cooperative interaction network. Notably, the Drude model enables enhanced engagement with catalytic residues in NtMGAM, whereas ligand binding in CtMGAM and α-amylase remains more flexible and less directly coupled to the catalytic machinery. These results highlight the importance of polarization in describing enzyme–ligand interactions and suggest that polarization can influence the balance and persistence of interactions in a system-dependent manner. These findings provide further computational support for the candidate inhibitors identified through computational screening.

## Graphical abstract

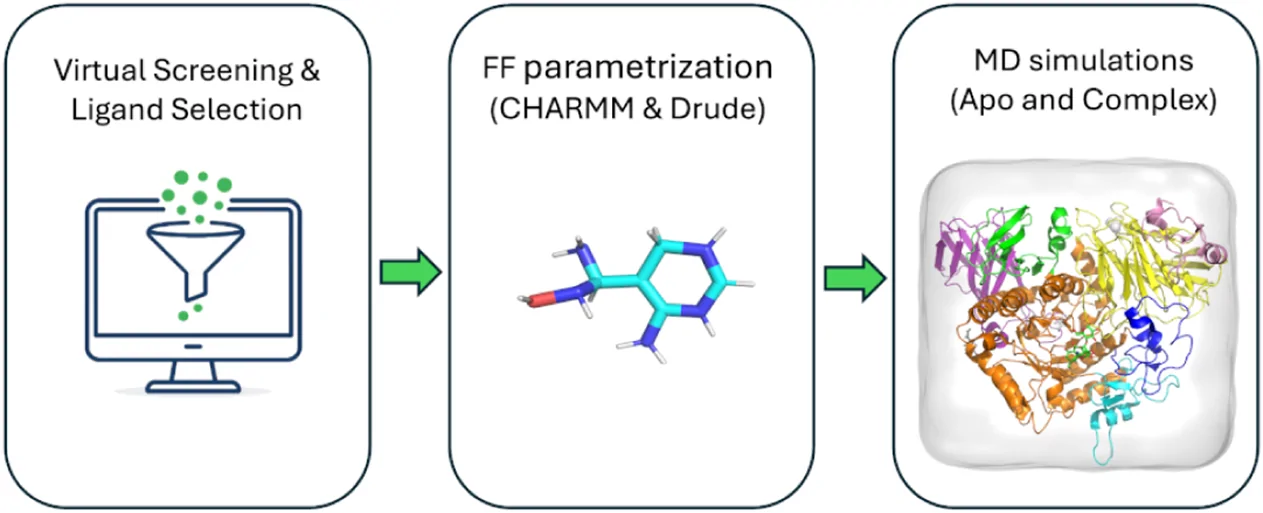

## Introduction

Enzymes responsible for the digestion of dietary carbohydrates play a fundamental role in human metabolism. Among these enzymes, pancreatic α-amylase and the intestinal brush-border enzyme maltase-glucoamylase (MGAM) are key contributors to the breakdown of starch into absorbable glucose molecules.^1^ Pancreatic α-amylase initiates the digestion of starch by cleaving internal α-1,4-glycosidic linkages within polysaccharides such as amylose and amylopectin, generating shorter oligosaccharides and maltodextrins.^2,3^ These products are subsequently hydrolyzed into glucose by exo-acting glucosidases located in the small intestine, including the N-terminal and C-terminal catalytic domains of MGAM (NtMGAM and CtMGAM, respectively).^1^ Because the activity of these enzymes directly influences postprandial glucose production,^4,5^ they have emerged as potential therapeutic targets for the management of type 2 diabetes. Inhibition of starch-digesting enzymes can slow carbohydrate breakdown and reduce glucose absorption, thereby helping regulate blood glucose levels after meals.^6–8^

Structural and biochemical studies have provided important insights into the architecture and catalytic mechanisms of these enzymes. High-resolution crystal structures have revealed that pancreatic α-amylase belongs to the glycoside hydrolase family 13 (GH13), characterized by a (β/α)_8_ barrel catalytic domain and a substrate-binding groove that accommodates multiple glucose units of the polysaccharide chain.^6^ In contrast, MGAM belongs to the glycoside hydrolase family 31 (GH31) and contains two distinct catalytic domains with overlapping but nonidentical substrate specificities.^9^ These structural studies have provided valuable information about the active sites and catalytic residues involved in carbohydrate hydrolysis. However, crystallographic structures represent static snapshots of proteins and do not fully capture the dynamic nature of enzyme conformations in solution. Protein flexibility can influence the accessibility of the binding pocket, and the stability of bound ligands, which are critical factors in inhibitor binding and enzyme function.^10,11^

Molecular dynamics (MD) simulations are a powerful computational approach for investigating the time-dependent behavior of biomolecular systems and complement experimental structural studies by revealing the dynamic motions of proteins at atomistic resolution. MD simulations can describe conformational fluctuations, structural stability, and intermolecular interactions over time. This approach has become widely used for studying enzyme flexibility, ligand binding stability, and the dynamic properties of protein active sites.^12^ In particular, MD simulations can help determine whether predicted binding pockets remain stable during the simulation and whether candidate inhibitors identified through virtual screening maintain favorable interactions with target proteins.

In our accompanying study,^13^ we identified potential inhibitors targeting pancreatic α-amylase, NtMGAM, and CtMGAM. SILCS-based pharmacophore modeling and virtual screening were employed to screen large chemical libraries and identify compounds with favorable predicted binding affinities. Representative high-scoring molecules were subsequently selected for further investigation. However, accurate MD simulations of ligand–protein complexes require reliable force field (FF) parameters for the ligand molecules. Because many drug-like compounds contain chemical functionalities that are not well represented within existing parameter databases, parameter refinement is often necessary to ensure that the molecular mechanics model accurately reproduces quantum mechanical (QM) properties of the ligand.

The CHARMM General Force Field (CGenFF) provides a framework for generating parameters for drug-like molecules based on analogy with previously parametrized chemical fragments.^14^ When parameters with high penalty scores are identified, additional refinement using QM calculations is required to ensure accurate representation of bonded and electrostatic interactions. To address this challenge, detailed CHARMM and Drude FF parametrization of the selected compounds is performed in this study.

Following parameter development, MD simulations were conducted to investigate both the intrinsic dynamics of the target enzymes and the stability of the predicted enzyme–ligand complexes. Simulations of the apo forms of NtMGAM, CtMGAM, and pancreatic α-amylase were first performed to characterize the baseline conformational behavior of the enzyme active sites in the absence of bound ligands. Because fluctuations in the geometry of enzyme binding pockets can influence ligand recognition and binding stability, analysis of pocket size during the simulation provides useful information about the structural stability of the catalytic cavity.

Subsequently, MD simulations of the enzyme–ligand complexes were performed using both CHARMM and Drude FFs. These simulations were aimed to evaluate the stability of ligand binding, characterize key intermolecular interactions between the ligands and active-site residues, and determine how ligand binding influences the conformational dynamics of the enzyme active site. Together, the analyses presented here provide a dynamic perspective on the behavior of the target enzymes and offer further computational characterization of the candidate inhibitors identified through the computational screening workflow.

## Methods

### Ligand Selection and Parametrization

The ligands identified in our accompanying study were MAYBRIDGE-SEW03510-3 for NtGMAM, CHEMBRIDGE-57665815-3 for CtMGAM, and CHEMBRIDGE-34773317-1 for α-amylase.^13^ The topology of each ligand was produced by CGenFF^14^ using the ligand structure in MOL2 format as input to obtain atom types, bonded terms, and preliminary partial atomic charges. CGenFF evaluates parameter reliability by assigning penalty scores to each bonded and electrostatic term.^15^ Parameters with large penalties from CGenFF were refined as described here prior to MD simulations of enzyme-inhibitor complexes. For Drude topologies, initial electrostatic and bonded parameters were assigned by analogy to other species in the FF and refined as described here.

### Quantum Mechanical Calculations

The first step in the parametrization procedure involved obtaining a reliable QM description of the ligand geometry. Geometry optimization was carried out to determine the lowest-energy configuration of each molecule. This optimized structure serves as the primary reference state for the entire parameter development workflow. Because molecules are expected to populate configurations close to their ground state during MD simulations, reproducing the properties of this optimized structure is a central objective of the FF parametrization process.

QM calculations were performed using Gaussian09 ^16^ for geometry optimizations, vibrational frequency calculations, and molecular dipole moments for CHARMM parametrization, or Psi4 ^17^ for single-point energy evaluations, molecular dipole moments for Drude parametrization, and molecular polarizability calculations. Geometry optimization and vibrational frequency calculations were performed using an MP2/6–31+G* model chemistry unless otherwise noted. For molecular dipole moments serving as target data for CHARMM parametrization, the MP2/6–31+G* model chemistry was used. For dipole moments, molecular polarizability, and single-point energy values used as target data for Drude parametrization, the RIMP2/aug-cc-pVQZ model chemistry^18^ was employed.

### Ligand–Water Interactions

Because most biomolecular simulations are performed in aqueous environments, it is important that the parametrized molecule interacts realistically with water, and such interactions are a foundational principle of the CHARMM and Drude FFs to balance electrostatic forces.^19,20^ For this reason, interactions between the ligand and individual water molecules were calculated using both QM and MM calculations to guide further refinement of electrostatic parameters.

QM calculations were performed on bimolecular complexes formed between the ligand and a single water molecule positioned near each of the chemically relevant interaction sites. These sites included not only functional groups capable of acting as hydrogen bond donors or acceptors, but hydrophobic and aromatic groups, as well. The resulting ligand–water complexes were subjected to QM optimizations with rigid geometries enforced for the ligand and water molecule, optimizing only the target water interaction distance. From these calculations, we determined both interaction energies (E_int_) and the distance at which the minimum E_int_ occurred (*r*_min_). When validating parameters for the Drude polarizable model, this MP2/6–31+G* distance was used directly as the target value. In contrast, for the CHARMM FF the target distance was adjusted by subtracting 0.2 Å from the QM-optimized value to compensate for the absence of polarization effects in the MM model, which are inherently present in the QM calculations.^19^

For the CHARMM FF, E_int_ between the ligand and water molecule were computed using the HF/6–3+G* model chemistry and subsequently multiplied by a scaling factor of 1.16 for neutral compounds before being used in the fitting procedure. The purpose of this correction is to account for the systematic underestimation of interactions such as dispersion and polarization in QM calculations, thereby aligning the computed energies with experimental or model data for more accurate FF development.^19^ For the Drude polarizable FF, ligand–water interaction energies were instead calculated using the RIMP2/aug-cc-pVQZ model chemistry without any scaling.

### Dihedral Potential Energy Surfaces

One-dimensional torsional energy scans were performed for selected dihedral angles within the ligand. These torsions were typically chosen based on high CGenFF penalty scores or because they corresponded to chemically important rotational degrees of freedom in the molecule. During the scan, the dihedral angle of interest was incrementally rotated from 0° to 345°, in increments of 15°, yielding 24 configurations. The target dihedral was held rigid while the remaining internal coordinates were allowed to relax. Single-point energies were calculated using the RIMP2/aug-cc-pVTZ and RIMP2/aug-cc-pVQZ model chemistries for the CHARMM and Drude FFs, respectively.

After obtaining the QM torsional energy profiles, the dihedral parameters in the FF were optimized to reproduce the reference energy surfaces. The fitting procedure allowed adjustable parameters to be systematically modified to minimize the difference between MM and QM energy surfaces. In the fitting procedure, the multiplicities and phase angles were predetermined based on similarity to existing FF parameters. Only phase angles of 0° or 180° were considered, which is standard for the CHARMM and Drude FFs. Only multiplicities of 1, 2, 3, and 6 were assigned.

### Electrostatic Parameter Fitting

After the QM target properties were obtained, electrostatic parameters were refined using a Monte Carlo/simulated annealing (MC/SA) optimization procedure, which iteratively adjusts selected parameters to reproduce target QM data.^21,22^ During optimization, atomic charges (and for the Drude model, atomic polarizabilities and Thole screening factors) were perturbed within specified windows. After each adjustment, properties were recalculated and compared to the QM target data. For CHARMM parametrization, the target data were the QM gas-phase dipole moment, multiplied by a factor of 1.15 to account for condensed-phase polarization effects. For Drude parametrization, the target data were the unscaled gas-phase dipole moment and molecular polarizabilities, scaled by a factor of 0.85.^20^

The quality of the fit was evaluated using the root-mean-square error (RMSE) between QM target values and FF-calculated properties. Parameter updates that reduce the RMSE are accepted, while those that increase it are conditionally accepted based on the Metropolis criterion. The optimization began at a high temperature (∼500 K) to allow broad exploration of parameter space and was gradually cooled, periodically scaling the temperature to 75% of its previous value, to refine the parameter set. This simulated annealing process continued until convergence was achieved (defined as an RMSE change <0.001 between two subsequent fitting steps).

### Dihedral Parameter Fitting

Optimization of the force constants was performed using the same MC/SA fitting procedure employed for electrostatic parameter refinement.^22^ In this approach, the dihedral force constants are treated as adjustable variables within the fitting program. The target data for the fitting procedure consisted of the QM torsional energies obtained from the potential energy scans, offset such that the global minimum corresponded to zero, allowing direct comparison with the energy profiles produced by the FF.

During the fitting process, force constants were iteratively adjusted until the MM torsional profile reproduced the major features of the QM potential energy surface. QM configurations with relative energies greater than 12 kcal mol^–1^ were excluded from the fitting procedure because such conformations correspond to highly strained geometries that are rarely sampled during MD simulations. The quality of the fit was evaluated using RMSE between all targeted energy values. For rotatable bonds connecting the subsections of the larger ligands, the dihedral parameters were optimized to reproduce the QM-optimized geometry of the complete ligand. The resulting parameters were evaluated by comparing the MM-minimized structure of the full ligand with its QM-optimized structure.

### Molecular Dynamics Simulations

MD simulations were performed for both the apo enzymes and the enzyme–ligand complexes. For the unbound systems, the available crystal structures were used as initial coordinates: NtMGAM (PDB: 3L4Y), CtMGAM (PDB: 3TOP), and pancreatic α-amylase (PDB: 4GQR). For the enzyme–ligand systems, the corresponding ligands were MAYBRIDGE-SEW03510-3 (NtMGAM), CHEMBRIDGE-57665815-3 (CtMGAM), and CHEMBRIDGE-34773317-1 (α-amylase), with ligand conformations obtained as described in the accompanying article.^13^ The initial pose of the ligands within the binding sites are shown in Figure 1.

**Figure 1:**
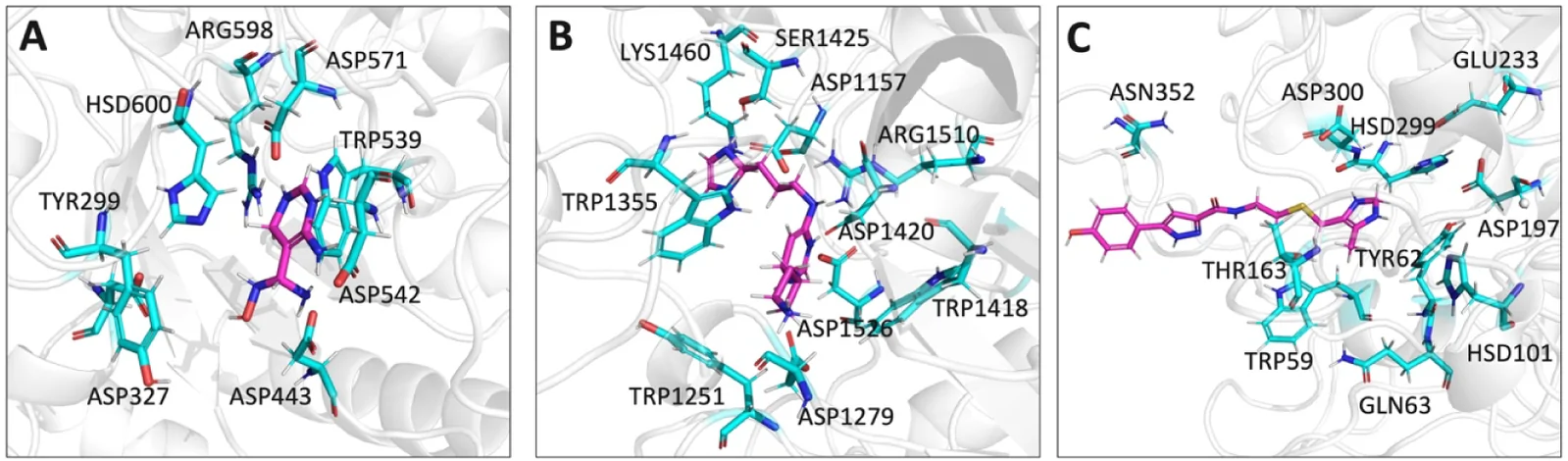
Initial binding poses of (A) MAYBRIDGE-SEW03510-3 in NtMGAM, (B) CHEMBRIDGE-57665815-3 in CtMGAM, and (C) CHEMBRIDGE-34773317-1 in pancreatic α-amylase. The displayed poses correspond to the lowest-energy conformations from SILCS-MC and were used as starting structures for MD simulations.

System preparation was initially carried out using the CHARMM molecular simulation package^23^ with CHARMM36 FF.^24^ Protonation states were assigned prior to solvation: His831 in NtMGAM was modeled in its ε-protonated (HSE) form to preserve the local hydrogen-bonding network. In α-amylase, Glu233 was modeled in its neutral (protonated) form to maintain coordination of the nearby chloride ion. The crystallographic Cl^–^ and Ca^2+^ ions present in PDB 4GQR were retained in all α-amylase simulations, including both apo and enzyme–ligand systems, using both the CHARMM and Drude FFs. The Ca^2+^ ion remained stably coordinated throughout the trajectories. Because the Cl^–^ ion is located near the catalytic site and forms interactions with Arg195, Asn298, and Arg337, its coordination environment was monitored throughout the simulations. Distances between Cl^–^ and these residues were calculated to assess differences in chloride coordination between the CHARMM and Drude FFs. For Arg195 and Arg337, the distance to Cl^–^ was calculated relative to the center of mass of the guanidinium group, whereas the corresponding distance for Asn298 was calculated using the polar side-chain group. Each enzyme or enzyme–ligand complex was then solvated in a cubic simulation box of TIP3P water molecules.^25–27^ Prior to the production simulations, the systems were subjected to energy minimization using CHARMM molecular simulation package^23^ to relieve unfavorable contacts generated during system construction. This minimization procedure consisted of 1000 steps of steepest descent followed by 1000 steps of adopted-basis Newton–Raphson minimization. Following minimization, the systems were equilibrated for 1 ns under an NPT ensemble using OpenMM 7.7.0.^28^ During this stage, position restraints were applied to all non-hydrogen atoms using a force constant of 500 kJ mol^–1^ nm^–2^ to maintain the overall structural integrity of the protein while allowing solvent relaxation. The temperature was maintained at 298 K using a Langevin thermostat, and the pressure was controlled at 1 atm using a Monte Carlo barostat.^29,30^ Periodic boundary conditions were applied in all three spatial dimensions. Short-range van der Waals forces were smoothly switched to zero between 10 and 12 Å, while long-range electrostatic interactions were evaluated using the particle mesh Ewald (PME) method with a real-space cutoff of 12 Å.^31^ Covalent bonds involving hydrogen atoms were constrained using the SHAKE algorithm, which enabled the use of a 2 fs integration timestep for the additive simulations.^32^

After completion of the CHARMM equilibration stage, the systems were converted to the Drude polarizable representation using CHARMM package.^23^ This conversion involved introducing Drude oscillators and associated lone-pair sites into the equilibrated structures. In addition, the TIP3P water molecules were replaced with the polarizable SWM4-HLJ water model.^33^ To relax the newly introduced Drude degrees of freedom, the systems were subjected to additional energy minimization consisting of 1000 steps of steepest descent followed by 500 steps of adopted-basis Newton–Raphson minimization. The polarizable systems were subsequently equilibrated under NPT conditions using a dual Langevin thermostat with Drude oscillators coupled to a low-temperature (1 K) relative thermostat. The barostat settings were the same as described for the additive simulations. The temperature was maintained at 298 K and the pressure at 1 atm. The treatment of nonbonded interactions was similar to the additive simulations, with the exception that the van der Waals potential (not forces) was switched off between 10 and 12 Å and electrostatic interactions calculated using the particle mesh Ewald (PME) method.^31^ The same position restraints applied during additive equilibration were also used for the polarizable simulations. Because the Drude model introduces additional high-frequency motions associated with the Drude particles, the integration timestep was reduced to 1 fs and covalent bonds involving hydrogen atoms were constrained. Each polarizable system was equilibrated for 1 ns prior to production simulations, which were carried out using OpenMM while maintaining the NPT ensemble and the thermostat and barostat parameters described above. To improve conformational sampling and assess the reproducibility of the simulations, three independent replicate simulations of 1 μs were performed using OpenMM for each apo and protein–ligand systems with both the CHARMM and Drude FFs. These simulations were initiated by assigning different randomly generated initial velocities at the start of the equilibration phase. The full, 1-μs production trajectories of apo enzymes and enzyme-ligand simulations were used for all structural and interaction analyses.

### Binding Pocket Volume Analysis

The stability and conformational variability of the catalytic binding pockets were evaluated by monitoring pocket volumes throughout the MD trajectories using the POVME (Pocket Volume MEasurer) software.^34^ Prior to pocket volume analysis, the MD trajectories were aligned to a common reference structure to remove global translation and rotation of the protein. Doing so places the protein structures in a common coordinate frame and facilitates consistent application of the predefined spatial region used to identify the binding pocket across all trajectory frames. The aligned trajectories were then exported as multi-frame PDB files, with solvent molecules removed, to serve as input for the POVME calculations.

For each enzyme system, the region corresponding to the active-site cavity was defined based on the location of the catalytic residues and the ligand-binding pocket observed in the crystal structures. Within this defined region, grid points that overlap with protein atoms were removed, leaving only those points that occupy the internal cavity of the binding pocket. Points located outside the relevant pocket boundaries or not contiguous with the primary cavity were also removed, resulting in a refined point cloud corresponding to the accessible pocket volume. The remaining grid points were then used to calculate the cavity volume for each frame of the trajectory.

## Results and Discussion

### Parametrization of the Predicted Inhibitors

The parametrization workflow described above was applied to three representative ligands selected from SILCS-based virtual screening: MAYBRIDGE-SEW03510-3 for NtMGAM, CHEMBRIDGE-57665815-3 for CtMGAM, and CHEMBRIDGE-34773317-1 for α-amylase. For each ligand, parameters were developed for both CHARMM and Drude FFs. The resulting topologies were evaluated based on their ability to reproduce QM target data, including electrostatic properties, ligand–water interactions, and dihedral potential energy surfaces.

Because the ligands selected for CtMGAM and α-amylase are relatively large, they were divided into three subsections to facilitate the parametrization process (Figure 2). For CHEMBRIDGE-57665815-3, all three subsections exhibited relatively high parameter and charge penalties, indicating that each region of the molecule required further parameter refinement. In contrast, for the α-amylase ligand CHEMBRIDGE-34773317-1, subsection 2 had low penalties, indicating that this region is already well represented within the existing CGenFF parameter space and therefore did not require additional refinement. The remaining subsections exhibited large penalties and were subjected to parameter optimization.

**Figure 2:**
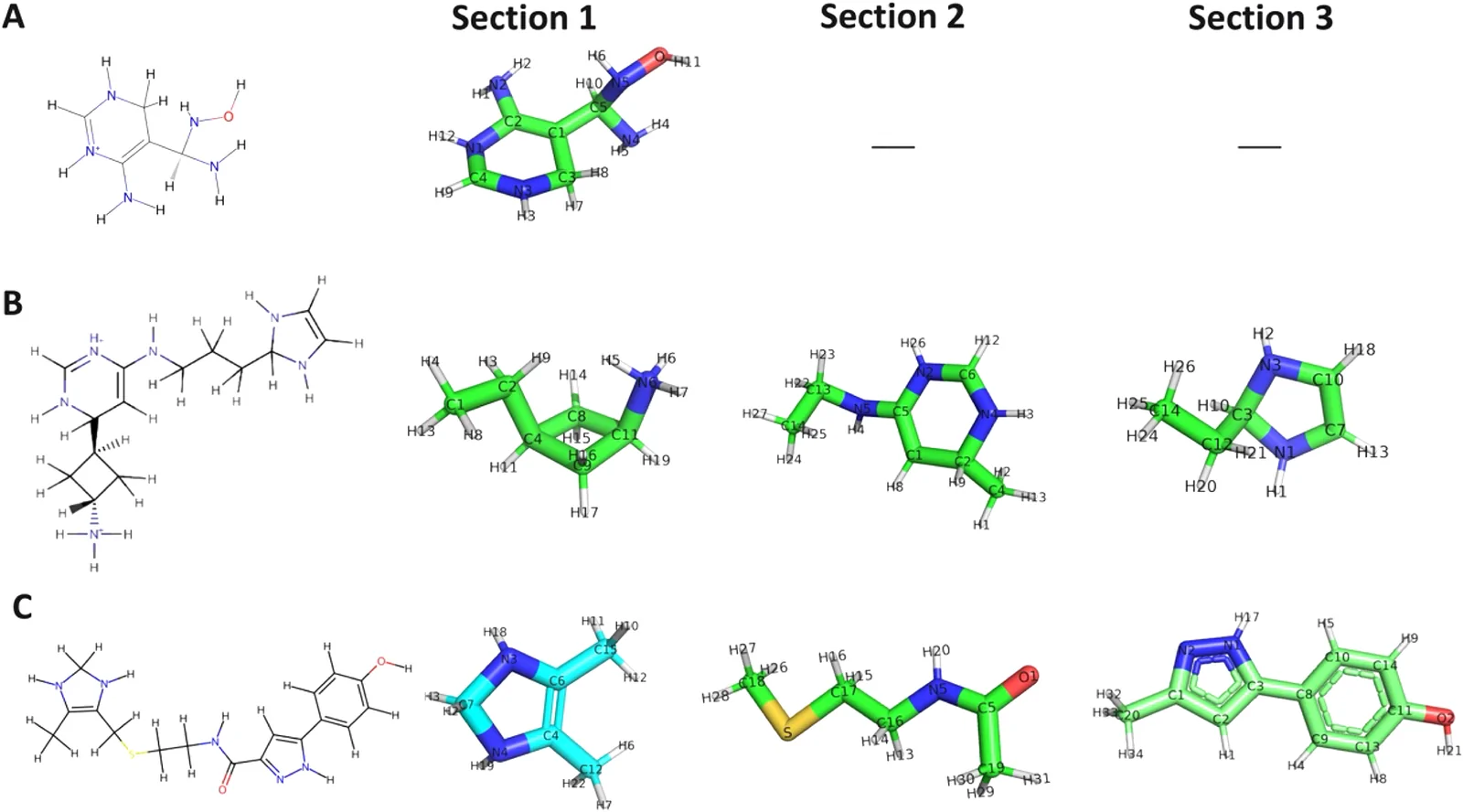
Two- and three-dimensional structures of the ligands selected for parametrization and subsequent MD simulations. (A) MAYBRIDGE-SEW03510-3 for NtMGAM, (B) CHEMBRIDGE-57665815-3 for CtMGAM, and (C) CHEMBRIDGE-34773317-1 for α-amylase. For the larger ligands in panels B and C, the molecule was divided into three subsections to facilitate the parameter fitting process.

The final comparison between the QM and MM dipole moment components for the CHARMM FF is summarized in the Supporting Information, Table S1. For all parametrized subsections, the optimized partial charges closely reproduced the target QM components of the dipole moment vector, indicating that these partial atomic charges provide a reasonable description of the electronic structure of the molecule.

For the Drude polarizable FF, the optimized parameters also reproduced the electrostatic properties well. The comparison of QM and MM dipole moment components is shown in the Supporting Information, Table S2, in which the dipole vector components obtained from the Drude FF closely match the QM target values across all subsections. In addition to the dipole moments, the Drude parametrization targeted the molecular polarizability tensors. As summarized in the Supporting Information, Table S3, the MM molecular polarizability tensor components and isotropic polarizabilities reproduce the QM target values well. These results indicate that both CHARMM and Drude models provide reasonable descriptions of the ligand charge distributions, while the Drude model additionally captures the molecular polarization response.

Ligand–water interaction energies and optimized geometries were calculated for representative hydrogen-bond donor and acceptor sites. Two possible interaction geometries were examined: 180° orientations, for a direct linear interaction, and out-of-plane orientations, representing interactions with lone-pair electron density on heteroatoms or aromatic systems. Representative examples of these interaction geometries are illustrated in the Supporting Information, Figure S1. The ligand–water interaction results for the three compounds using CHARMM and Drude FFs are summarized in the Supporting Information, Tables S4–S9. For each interaction, *r*_min_ and E_int_ from the QM calculations were compared with the corresponding values calculated using the FF. Overall, the MM interaction energies reproduced the QM target values well across the examined interaction sites, with the Drude FF performing slightly better across all molecules.

To further refine the bonded parameters of the ligands, dihedral potential energy surfaces were calculated for selected torsional angles that either exhibited large penalty scores in the initial CGenFF parameter assignment or were associated with key rotatable bonds that would influence the overall conformation of the molecules. The resulting MM potential energy surfaces using both CHARMM and Drude FFs were compared with those obtained from the QM calculations, and torsional parameters were adjusted where necessary to improve agreement between the energy profiles. For the α-amylase ligand (CHEMBRIDGE-34773317-1), subsection 2 had a parameter penalty score of zero, indicating that the corresponding parameters were already encompassed by the existing CGenFF parameter space and needed no refinement. In the case of subsection 3 of the CtMGAM ligand (CHEMBRIDGE-57665815-3), the very small parameter penalty led us to perform an additional validation step instead of immediately proceeding with dihedral parameter optimization. Specifically, the MM structure was energy minimized and subsequently compared with the QM-optimized geometry. As shown in Figure 3, the minimized MM structure reproduced the QM geometry with excellent agreement, indicating that the existing parameters already provide an adequate description of the conformational preferences in this portion of the molecule. Based on this observation, no further dihedral adjustments were performed for subsection 3 during the CHARMM parametrization.

**Figure 3:**
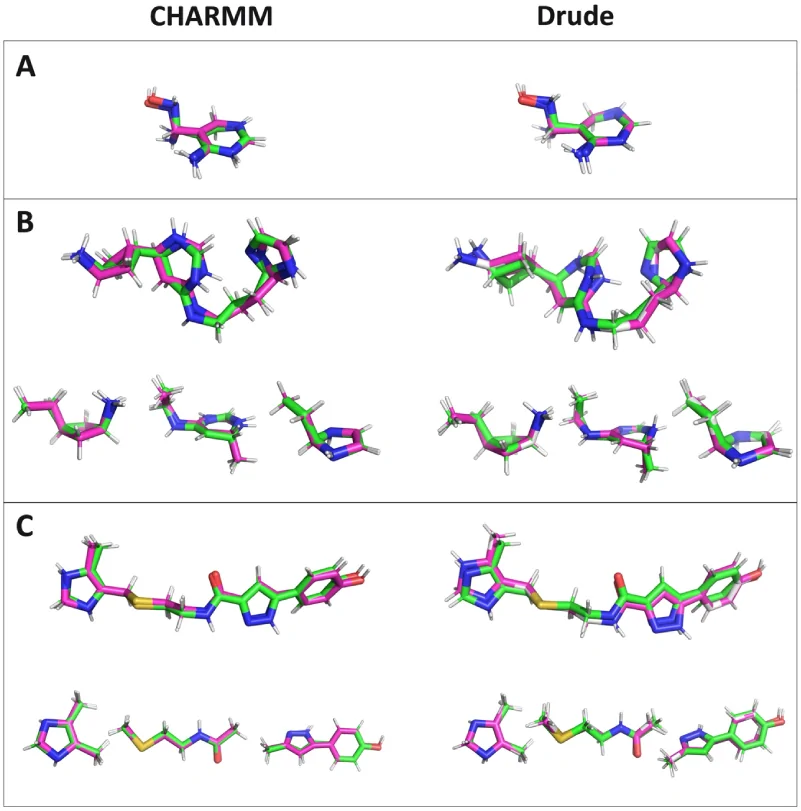
Comparison between QM-optimized and MM-minimized geometries of the parametrized ligands. The QM-optimized structures are shown with green carbon atoms, while the MM-minimized structures are shown with purple carbon atoms. (A) MAYBRIDGE-SEW03510-3, identified for NtMGAM, (B) CHEMBRIDGE-57665815-3, identified for CtMGAM, with the three subsections used during parametrization, and (C) CHEMBRIDGE-34773317-1, identified for α-amylase, with the three subsections.

For the remaining subsections and ligands, torsional parameters were optimized to reproduce the QM potential energy surfaces. Comparisons between the QM and MM energy profiles generated using CHARMM and Drude FFs are shown in the Supporting Information, Figures S2–S7. The optimized torsional parameters successfully reproduced the shape, minima, and relative barrier heights of the QM energy surfaces, demonstrating that the resulting FF parameters provide a reliable description of ligand conformational energetics.

### Structural Characterization of Apo Enzymes

MD simulations were performed for the apo forms of the enzymes to examine their intrinsic structural stability and dynamic behavior in the absence of bound ligands. The full, 1-μs production trajectories of the three replicates were used for all structural and interaction analyses. These simulations provide a baseline for evaluating conformational flexibility, particularly in loop regions surrounding the catalytic pocket, which may influence the accessibility and shape of the binding site. The apo systems were therefore analyzed to identify flexible regions that could contribute to changes in pocket geometry during the simulations. NtMGAM and CtMGAM both contain a catalytic domain that adopts a (β/α)_8_ barrel fold that is characteristic of glycoside hydrolases. In addition to the catalytic core, these enzymes include two inserted loop regions, referred to as inserted loop 1 and inserted loop 2, located adjacent to the catalytic site that can influence the accessibility and volume of the binding pocket. In contrast, pancreatic α-amylase consists of three major structural domains, designated Domains A, B, and C. Domain A forms the catalytic core of the enzyme and contains the active-site residues. Domain B is located adjacent to Domain A and contributes to the formation of the catalytic pocket, while Domain C is positioned at the C-terminus and primarily contributes to maintaining the structural integrity of the enzyme. The structural organization of these enzymes is shown in Figure 4. This structural division serves as the basis for the domain-specific root-mean-square deviation (RMSD) and root-mean-square fluctuation (RMSF) analyses presented in the following sections.

**Figure 4:**
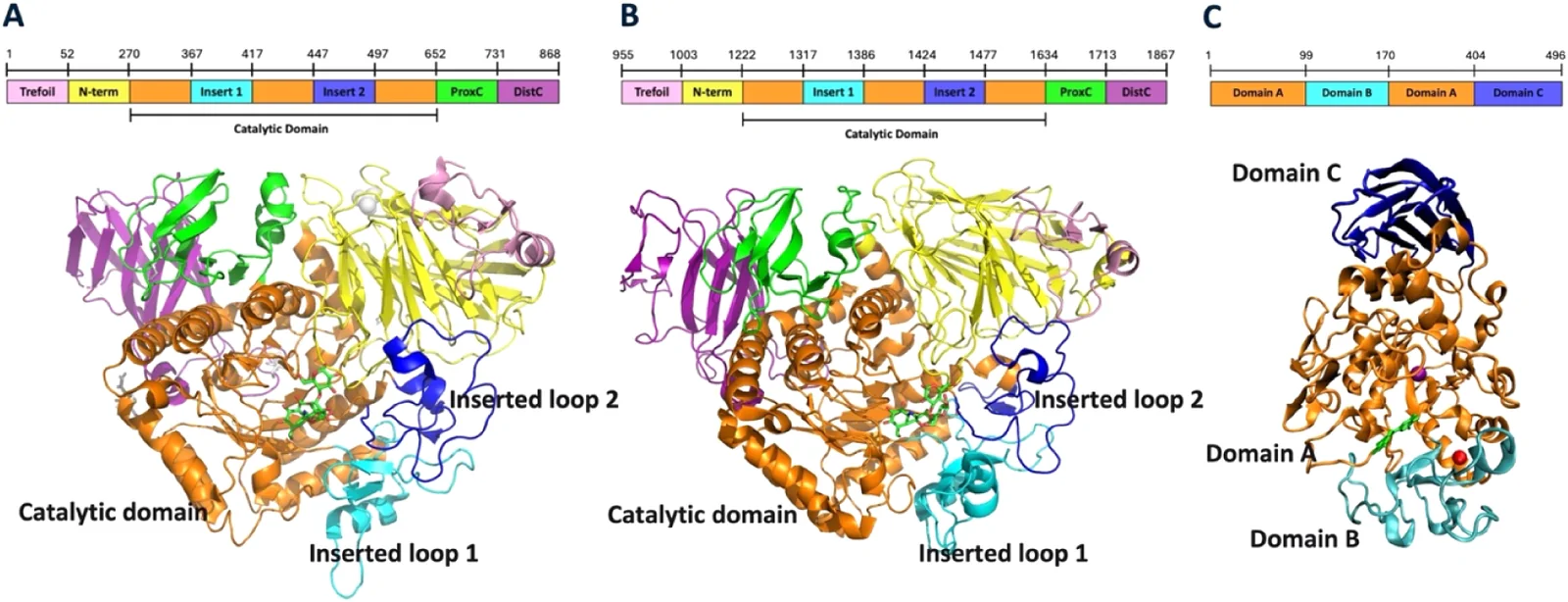
Structural organization of the enzymes studied in this work. (A) NtMGAM, (B) CtMGAM, and (C) pancreatic α-amylase.

RMSD and RMSF analyses were performed using AmberTools23.^35^ The structural stability of the apo NtMGAM was evaluated by calculating the RMSD of Cα atoms throughout the simulations. RMSD values were computed for the entire enzyme as well as for individual structural regions labeled in Figure 4. For whole-enzyme RMSD, trajectories were aligned using Cα atoms of the full enzyme. For domain- or region-specific RMSD analyses, trajectories were aligned using the Cα atoms of the corresponding structural region prior to calculating its RMSD. RMSD profiles obtained from simulations performed using the CHARMM and the Drude FFs are shown in the Supporting Information, Figure S8. For both FFs, the RMSD of the Cα atoms for the entire enzyme remained relatively low and stable over the course of the simulations, indicating that the overall structure of NtMGAM was well maintained. The average RMSD values of the entire enzyme calculated from the three replicate simulations were 2.0 ± 0.3 Å and 2.0 ± 0.2 Å for the CHARMM and Drude simulations, respectively, where the reported uncertainties represent standard deviations. These values indicate that the global structure of the enzyme was preserved to a similar extent with both FFs.

To further examine local structural fluctuations, RMSD values were calculated separately for the catalytic domain and the inserted loop regions, which have been reported to influence the size and accessibility of the active-site pocket. The catalytic domains remained stable in both CHARMM and Drude simulations, with values generally ranging between 1–2 Å. However, one replicate in each model exhibited increased fluctuations in this region. Inserted loop 1 showed less structural deviation in the Drude simulations, whereas inserted loop 2 behaved similarly in both models, with RMSD values ranging from ∼0.75 to 1.5 Å. Within the NtMGAM system, the structure of the N-terminal region deviated less in the Drude simulations compared to the CHARMM simulations.

To characterize the flexibility of NtMGAM during the simulations, the RMSF of Cα atoms was averaged over the three replicates for both the CHARMM and Drude simulations. The RMSF values were mapped onto the surface representation of the enzyme to visualize the spatial distribution of residue fluctuations (Supporting Information, Figure S9). Regions surrounding the active site in the Drude simulations had lower RMSF values, indicating reduced flexibility. In contrast, the CHARMM simulations yielded increased flexibility in the N-terminal region near the active site. Additionally, part of inserted loop 1 in the CHARMM simulations had increased flexibility, but this region is distal to the active site. Despite these minor differences, in both CHARMM and Drude simulations the catalytic domain remained relatively stable throughout the simulations, suggesting that the core structural framework of the enzyme is preserved.

The same analysis was performed for apo CtMGAM. Across the three replicate simulations, the average RMSD of the full enzyme was 2.6 ± 0.4 Å and 2.6 ± 0.3 Å for the CHARMM and Drude simulations, respectively, demonstrating comparable global stability between the two FFs. To identify regions with greater conformational deviation, RMSD values were evaluated separately for key structural elements of the enzyme, including the catalytic domain, inserted loop 1, inserted loop 2, and the N-terminal region (Supporting Information, Figure S10). Among these regions, inserted loop 1 in the CHARMM simulations exhibited the largest deviations from the starting structure, indicating substantial conformational rearrangements during the simulations. Inserted loop 2 in the CHARMM trajectories also showed elevated RMSD values, suggesting increased flexibility relative to other regions of the enzyme. In contrast, the catalytic domain remained comparatively stable in both CHARMM and Drude simulations, with particularly consistent behavior observed in the Drude model.

For CtMGAM, the RMSF mapping identified inserted loop 1 as the most flexible region of the enzyme in the CHARMM simulations (Supporting Information, Figure S11). However, its flexibility was noticeably lower in the Drude simulations. In the Drude model, other structural elements, including the catalytic domain, exhibited relatively low fluctuations, indicating that the core architecture of CtMGAM remained well preserved. In contrast, the CHARMM simulations produced moderate flexibility in the catalytic domain.

In the CHARMM simulations of pancreatic α-amylase, the average RMSD values of Domains A, B, and C were 1.8 ± 0.4 Å, 1.8 ± 0.6 Å, and 1.2 ± 0.3 Å, respectively (Supporting Information, Figure S12). Simulations performed with the Drude FF produced lower deviations overall, with corresponding RMSD values of 1.6 ± 0.2 Å, 1.2 ± 0.2 Å, and 1.2 ± 0.3 Å. Residue-level flexibility was similarly examined by calculating the RMSF of Cα atoms. The RMSF mapping reveals that Domain A had the largest fluctuations in CHARMM simulations, with particularly high RMSF observed for residues 305–308 and 346–352, which are located within loop regions of Domain A, whereas the Drude simulations exhibited comparatively lower flexibility in this region (Supporting Information, Figure S13). Other parts of the enzyme, including Domain C, remained relatively rigid throughout the trajectories. Together, these results indicate that the global architecture of α-amylase was well preserved with both FFs. However, the Drude simulations produced greater structural consistency throughout the trajectories, with reduced fluctuations in the binding site compared to CHARMM.

Because the Cl^–^ion is positioned within the α-amylase active site, we next examined its behavior and local coordination environment to determine whether the differences in conformational dynamics between the two FFs were accompanied by changes in chloride interactions. The Cl^–^ ion remained associated with the binding site throughout all three Drude trajectories and two of the three CHARMM trajectories, with dissociation occurring at approximately 730 ns in the remaining CHARMM trajectory. To characterize the Cl^–^ coordination environment, distances between Cl^–^ and Arg195, Asn298, and Arg337 were monitored throughout the simulations. In the apo simulations, the Cl^–^–Arg195 distance was 3.9 ± 0.3 Å in CHARMM and 5.1 ± 0.8 Å in Drude, indicating a less proximal Arg195 interaction in the Drude simulations. The Cl^–^–Arg337 distance was similar between CHARMM (4.2 ± 0.5 Å) and Drude (4.0 ± 0.3 Å), as was the Cl^–^–Asn298 distance, which was 7 ± 2 Å in CHARMM and 8 ± 2 Å in Drude. These results indicate that the overall chloride coordination environment was broadly similar between the FFs, with the largest difference occurring in the Cl^–^–Arg195 interaction.

### Binding Pocket Volume Analysis of Apo Enzymes

The dynamic behavior of the catalytic pockets during the apo enzyme MD simulations was further evaluated by calculating the binding pocket volumes using POVME. As noted above, several loop regions located near the catalytic pockets, particularly inserted loop 1 in both MGAM domains and Domain A in α-amylase, exhibited increased flexibility during the simulations. Because motions in these regions can influence the size and accessibility of the catalytic cavity, monitoring the pocket volume provides additional insight into how these structural fluctuations affect the active-site geometry and accessibility.

Pocket volumes were computed for each frame of the MD trajectories for the three replicate simulations performed using both CHARMM and Drude FFs. The resulting average pocket volumes across the replicate simulations and the distribution of pocket volumes sampled during the three trajectories are tabulated in the Supporting Information, Table S10, and illustrated in Figure S14. For each enzyme system, the reported mean pocket volume was calculated from the three replicate trajectories, with the standard deviation calculated from the replicate values.

For NtMGAM, the average pocket volumes were 638 ± 110 Å^3^ and 572 ± 68 Å^3^ for the CHARMM and Drude simulations, respectively. The distributions indicate a broader range of sampled pocket volumes in the CHARMM simulations compared to the Drude simulations, suggesting that the NtMGAM binding pocket underwent larger fluctuations in the CHARMM FF trajectories. This behavior is consistent with the RMSF analysis, in which inserted loop 1 and the N-terminal region exhibited increased flexibility in the CHARMM simulations, which may contribute to the larger variation in pocket volume. For CtMGAM, the catalytic pocket was considerably larger than that of NtMGAM. The average pocket volume obtained from the CHARMM simulations was 1161 ± 39 Å^3^, whereas the Drude simulations produced a slightly larger average value of 1258 ± 191 Å^3^. The broader spread of pocket volumes in the Drude simulations was primarily driven by the larger pocket volume (1472.3 Å^3^) observed in one of the replicate trajectories. Aside from this replicate, the remaining trajectories produced pocket volumes similar to those observed in the CHARMM simulations, suggesting that the active-site volume is comparable with both FFs. Thus, the difference in the mean pocket volumes appears to reflect variability among the Drude replicates rather than a consistent difference in pocket size between the two FFs. For pancreatic α-amylase, the average pocket volumes for CHARMM and Drude simulations were 684 ± 226 Å^3^ and 579 ± 94 Å^3^, respectively. These volumes are consistent with the RMSF analysis, which showed greater flexibility in Domain A for the CHARMM simulations, a region located near the catalytic site and therefore likely to influence pocket geometry.

The time-dependent pocket volume profiles show that the volumes for all three enzymes stabilized as the simulations progressed (Figure S15). The NtMGAM and α-amylase CHARMM simulations exhibited larger variations in pocket volume compared to the corresponding Drude simulations. This observation is consistent with the RMSD and RMSF results, which also indicated increased variability in specific regions of the enzymes in the CHARMM simulations. The RMSD, RMSF, and pocket volume analyses indicate that the apo forms of the enzymes maintain stable global structures throughout the simulations while exhibiting localized flexibility in specific loop regions surrounding the catalytic pockets. Fluctuations in the inserted loop regions of both MGAM domains and Domain A of α-amylase contributed to moderate variations in the active-site volumes, suggesting an opportunity for specific targeting based on ligand size. These results provide a structural baseline for understanding the intrinsic dynamics of the enzymes in the absence of ligands. Building on this foundation, MD simulations of the enzyme–ligand complexes were subsequently performed to investigate how ligand binding influences the stability of the catalytic pockets and to examine the interactions between the identified inhibitor candidates and their respective enzyme targets.

### Molecular Dynamics of NtMGAM–Ligand Complex

MD simulations of the NtMGAM–ligand complex were performed using both CHARMM and Drude FFs with the primary goal of assessing whether the ligand remains stably bound within the catalytic pocket over the course of the simulations. Analysis of the trajectories show that the protein backbone remains stable throughout the trajectories, with average RMSD values of 2.2 ± 0.6 Å and 2.0 ± 0.2 Å for the CHARMM and Drude simulations, respectively, confirming that ligand binding did not impact the overall fold of the enzyme (Supporting Information, Figure S16). Domain-specific analysis further showed that the structure of the catalytic domain remained intact (RMSD of 1.3 ± 0.3 Å in CHARMM and 1.4 ± 0.3 Å in Drude), while loop regions near the active site deviated somewhat in structure. Per-residue RMSD analysis identified residues 327–333, which form a loop region, as the residues with the highest RMSD within the catalytic domain. In addition, inserted loop 1 deviated more in the CHARMM simulations (2.1 ± 0.4 Å) compared to the Drude simulations (1.5 ± 0.4 Å), whereas inserted loop 2 behaved similarly in both CHARMM and Drude simulations (∼1 Å). Thus, the higher RMSD observed in the ligand-bound simulations was mainly associated with local loop and replicate-to-replicate variability, consistent with the behavior observed in the apo simulations, rather than a distinct structural rearrangement of the catalytic domain. RMSD analysis of the heavy atoms in ligand show that the ligand remains stably bound within the catalytic pocket in both FFs, with RMSD values equal to 0.27 ± 0.07 Å and 0.9 ± 0.2 Å in CHARMM and Drude, respectively (Figure 5). The relatively low and stable ligand RMSD values indicate that the ligand remained close to its initial binding pose throughout the simulations. Previous molecular dynamics studies have similarly used ligand RMSD and its temporal stability as an indicator of pose persistence, with correctly placed ligands generally showing more stable trajectories than incorrectly placed poses.^36,37^ In the present study, the ligand RMSD remained relatively stable in both FFs, supporting retention of the screened pose over the simulated time scale, although ligand RMSD alone does not establish the correctness of the pose or experimental binding affinity.

**Figure 5:**
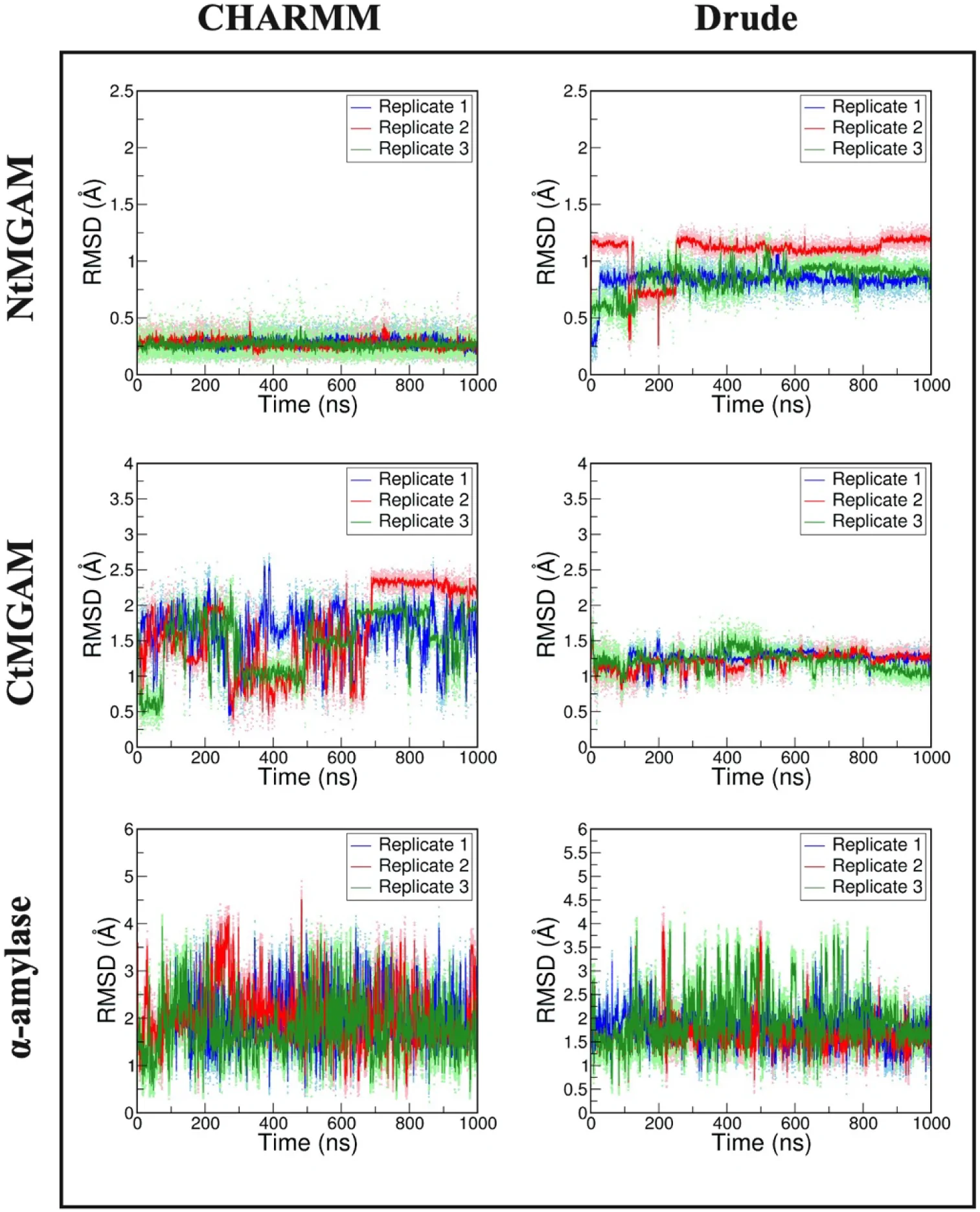
Ligand RMSD in complex with NtMGAM, CtMGAM, and α-amylase.

To characterize the persistence of polar interactions that contribute to ligand binding to the enzyme, hydrogen-bond occupancies were calculated over the course of the simulations. This analysis revealed distinct differences in how the ligand interacted with the active site in the CHARMM and Drude FFs. In the CHARMM simulations, ligand binding was dominated by a single strong interaction with Asp443 (∼89% occupancy), with only minor contributions from other residues (Figure 6). In contrast, the Drude simulations exhibited a dynamic hydrogen-bonding network involving multiple residues, including Asp542, His600, and Tyr299. Rather than relying on a single dominant interaction, the ligand in the Drude model formed multiple simultaneous or transient interactions, reflecting a more heterogeneous and adaptable interaction environment within the active site, which is also reflected in the slightly higher RMSD of the ligand compared to the CHARMM simulations.

**Figure 6:**
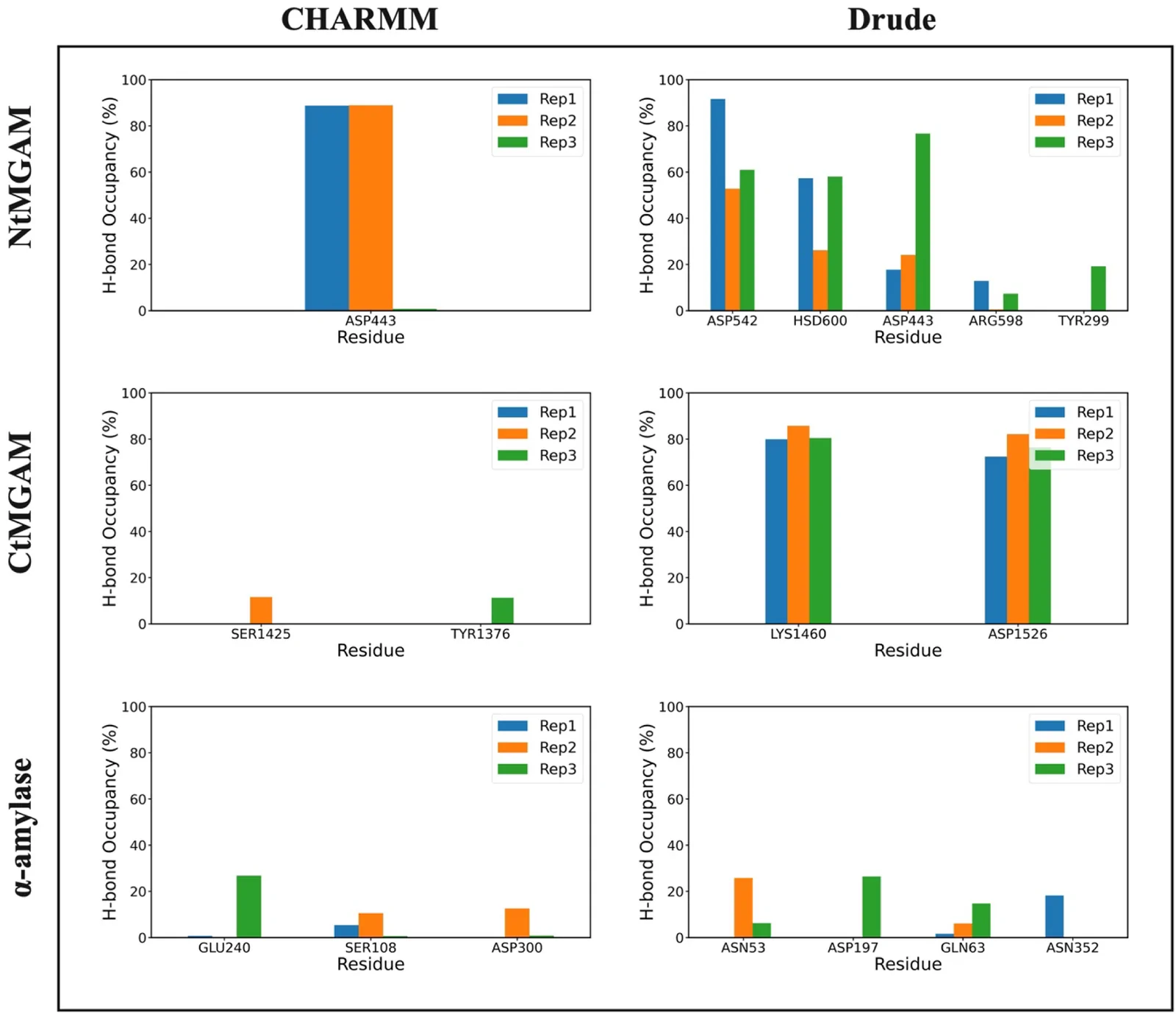
Hydrogen-bonding interactions between ligands and NtMGAM, CtMGAM, and α-amylase, reported as hydrogen bond occupancy (%) in simulations using the CHARMM and Drude FFs.

To complement this analysis and capture other ligand–protein interactions including hydrophobic and van der Waals contacts, we conducted contact frequency analysis to identify residues that remained close to the ligand throughout the simulation. A contact was defined as any heavy atom of the ligand being within 4 Å of any heavy atom of the residues in enzyme. This analysis is particularly useful for identifying persistent binding-site residues even when hydrogen bonds are weak or transient. In the CHARMM simulations, the ligand maintained persistent contacts with residues such as Asp443, His600, and Trp441 with contact frequencies above 90% across all three replicates. Additional residues such as Tyr299 also showed high and reproducible contact frequencies, although some variability was observed for residues such as Trp539 across replicates. These results indicate a relatively stable and well-defined interaction network when using the CHARMM FF (Figure 7). In contrast, the Drude simulations revealed a broader and more distributed set of high-frequency contacts. Residues such as Phe575, His600, Asp571, Trp539, and Gly541 consistently exhibited very high contact frequencies (typically >95–99%) across replicates, while additional residues (e.g., Asp542 and Asp443) contributed more variably to ligand stabilization. Compared to CHARMM, the Drude model captured a greater number of interactions with high persistence, suggesting that ligand binding is stabilized by a more robust and consistently maintained interaction network.

**Figure 7:**
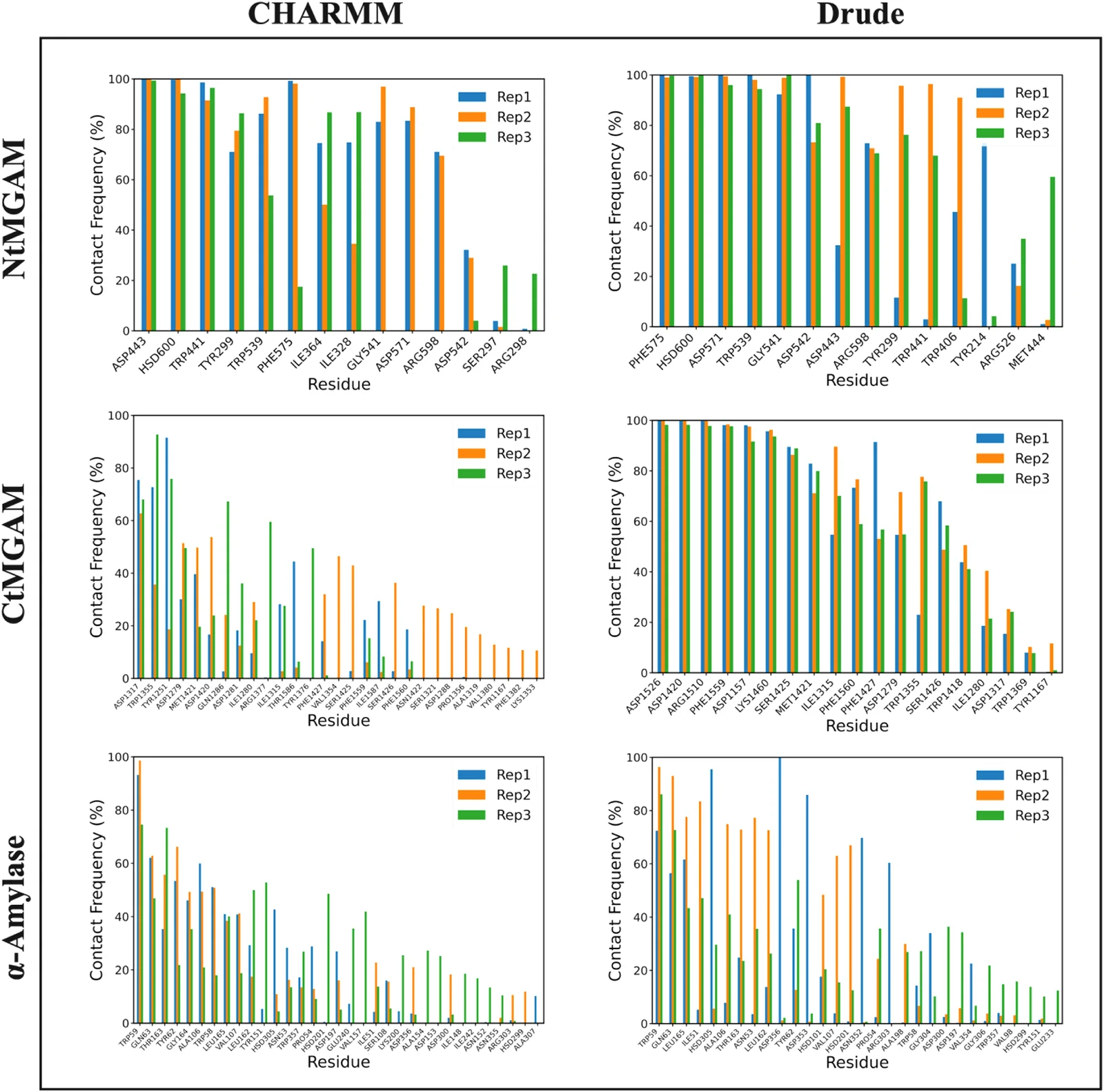
Ligand–protein contact frequency between ligands and NtMGAM, CtMGAM, and α-amylase, defined as contacts within ≤4 Å and reported as the percentage of simulation frames exhibiting such interactions, for both CHARMM and Drude simulations.

To directly assess whether the ligand engaged the catalytic residues (Asp443 and Asp542), the minimum distances between the ligand and the carboxylate oxygen atoms in catalytic residues were monitored throughout the simulations. In the CHARMM simulations, the ligand remained closely associated with the nucleophilic residue Asp443 (∼2.7 Å), while interacting more weakly with the acid/base residue Asp542 (∼4.2 Å), indicating that the binding mode is primarily centered around the nucleophile (Supporting Information, Figure S17). In contrast, in the Drude simulations, the ligand frequently adopted poses that that interacted more symmetrically with both catalytic residues (∼3 Å).

The simulations of NtMGAM in complex with MAYBRIDGE-SEW03510-3 demonstrate that the ligand remains stably bound within the NtMGAM catalytic pocket in both FFs, while revealing notable differences in the underlying interaction patterns. The CHARMM FF suggests a binding mode dominated by a single interaction with the nucleophilic residue, whereas the Drude FF captures a more distributed interaction network that included both catalytic residues. This behavior may reflect the ability of the Drude model to respond to changes in the local electrostatic environment, allowing the ligand to adapt its interactions within the binding site while remaining stably associated with the enzyme. Although NtMGAM has the smallest active-site volume among the enzymes studied here (Supporting Information, Figure S15 and Table S10), the Drude FF showed the ability to allow the ligand to sample additional interactions within the catalytic pocket while maintaining a stable association with the enzyme. In such a small volume, it is likely that the use of the CHARMM FF caused the ligand to remain somewhat restricted and biased toward its initial pose. The softness of the interactions with the Drude FF thus promotes subtle configurational changes that may be important for understanding mechanisms of inhibition.

### Molecular Dynamics of CtMGAM–Ligand Complex

In both CHARMM and Drude simulations, the average backbone RMSD values were 2.9 ± 0.5 Å and 2.7 ± 0.3 Å in CHARMM and Drude, respectively, indicating that the global fold was preserved throughout the simulations (Supporting Information, Figure S18). However, compared to the NtMGAM system, CtMGAM deviated more from its initial structure.

The positional variability of the ligand in this system was evaluated using RMSD of ligand heavy atoms, yielding values of 1.6 ± 0.5 Å in CHARMM and 1.2 ± 0.1 Å in Drude (Figure 5). This outcome indicates that the ligand remained confined within the active site and did not dissociate during the simulations, with the Drude simulations showing somewhat lower positional variability than CHARMM. Compared to NtMGAM, the higher RMSD values obtained in the CtMGAM-ligand systems suggest greater conformational freedom, but in this system, the binding pose was slightly better maintained by the Drude FF.

In contrast to the NtMGAM system, hydrogen-bonding interactions were weaker and less persistent in the case of CtMGAM. In the CHARMM simulations, no single residue consistently dominated ligand interactions, reflecting a lack of stable, long-lived hydrogen bonds (Figure 6). The Drude simulations, however, revealed more prevalent interactions, particularly involving residues such as Lys1460 and Asp1526, which exhibited high occupancies. This outcome suggests that polarization effects enable stronger and more consistent electrostatic interactions, even within a dynamic binding environment.

Contact frequency analysis highlights differences between the two FFs. In the CHARMM simulations, ligand contacts with residues such as Tyr1251, Trp1355, and Asp1317 were variable across replicates, reflecting a more transient interaction pattern (Figure 7). In contrast, the Drude simulations showed a stable and persistent contact network, with residues including Asp1526, Asp1410, Arg1510, Phe1559, Asp1557, and Lys1460 maintaining contact (>95%) with the ligand across all replicates. These results indicate that the ligand in Drude system remained well stabilized in the active site.

Analysis of interactions with catalytic residues revealed a key difference from the NtMGAM system. In the CHARMM CtMGAM simulations, the ligand did not remain in proximity to either the nucleophilic residue Asp1420 or the acid/base residue Asp1526, suggesting limited engagement with the catalytic residues (Supporting Information, Figure S20). In contrast, in the Drude simulations, the ligand interacted closely with both residues, with distances of ∼3 Å to carboxylate oxygen atoms. This outcome indicates that the ligand remained tightly anchored to the catalytic residues and may be capable of inhibiting enzyme activity more efficiently than would be predicted by the CHARMM FF.

Both models indicate that the CHEMBRIDGE-57665815-3 ligand remained in complex with the CtMGAM active site throughout the simulations. However, unlike the NtMGAM system, ligand stabilization in CtMGAM using CHARMM FF was characterized by greater conformational flexibility and weaker hydrogen-bonding interactions. As such, the ligand predominantly occupied a more dynamic and less tightly defined binding mode within the active site. The Drude FF, on the other hand, predicted that the ligand engages in strong electrostatic interactions that stabilized association with catalytic residues. Given that CtMGAM has the largest active site volume (Supporting Information, Figure S15 and Table S10), the difference in results between the two FFs suggests that CHARMM enables broader sampling based on attraction to more distal enzyme residues, whereas the Drude FF maintains strong interactions, even when competing interactions may be available throughout the larger volume.

### Molecular Dynamics of α-Amylase–ligand Complex

In simulations of α-amylase with both FFs, the overall enzyme structure was preserved, but the Drude simulations had consistently lower RMSD values (1.9 ± 0.2 Å) than the CHARMM simulations (2.4 ± 0.4 Å), indicating a slightly greater preservation of initial structure (Supporting Information, Figure S19). The same trend is apparent at the domain level, where Domains A, B, and C all exhibited lower RMSD values in the Drude model. This difference is especially notable for Domain B, whose RMSD was 3.1 ± 1.1 Å with CHARMM and 1.5 ± 0.2 Å with Drude. Since Domain B lies adjacent to the catalytic pocket and contributes to substrate recognition, these results indicate that the Drude FF more faithfully models a key binding environment than CHARMM. However, although Domain B was modeled more accurately with the Drude FF, the ligand pose was similarly preserved with both FFs, with RMSD 1.9 ± 0.5 Å with CHARMM and 1.8 ± 0.4 Å with Drude. As a result, the ligand remained bound in the active site throughout the simulations (Figure 5), indicating retention of the simulated binding pose over the 1-μs trajectories.

Hydrogen-bonding interactions in both CHARMM and Drude systems were generally weak and transient (Figure 6). In the CHARMM simulations, multiple residues contributed modestly to ligand interactions, but no single residue exhibited consistently high occupancy. A similar pattern was observed in the Drude simulations, for which interactions were distributed across several residues rather than concentrated at a specific site. This behavior suggests that despite the presence of charged and polar residues in the active site, persistent hydrogen bonding between the ligand and enzyme was not observed, which may be related to the greater conformational freedom of the compound within the binding site, as indicated by its higher RMSD values relative to the other two compounds in NtMGAM and CtMGAM.

Contact frequency analysis provided further insight into ligand stabilization. In the CHARMM simulations, residues such as Trp59, Gln63, Thr163, and Tyr62 maintained contacts with the ligand, although these interactions varied in persistence (Figure 7). In contrast, the Drude simulations had more consistent contact patterns, with residues including Trp59, Gln63, and Leu165 maintaining relatively higher contact frequencies across simulations. These residues are located within Domains A and B, confirming that the ligand remained positioned within the active site. The overall interaction pattern indicates that ligand stabilization in the α-amylase active site is primarily driven by hydrophobic and van der Waals interactions rather than strong, directional hydrogen bonds.

Distances between the ligand and the catalytic residues Asp197 (nucleophile) and Glu233 (general acid) were analyzed to assess direct engagement with the catalytic residues (Supporting Information, Figure S21). In the CHARMM simulations, the ligand remained relatively far away from both residues, with average distances of ∼6.8 Å for Asp197 and ∼11.6 Å for Glu233. Although occasional close contacts (∼2.4–3.5 Å) were observed, these interactions were transient. A similar behavior was observed in the Drude simulations. The ligand remains separated from Asp197 and Glu233, with average distances of 6.7 Å and 9.6 Å, respectively. While the Drude model allowed for somewhat closer sampling of these key interactions, particularly for Glu233, these interactions were not sustained. These results indicate that the ligand does not consistently engage the catalytic residues in α-amylase, suggesting that ligand binding is stabilized within the broader active-site region rather than through direct interaction with the catalytic residues.

Given the proximity of Cl^–^ ion to the catalytic residues, we next examined whether ligand binding was associated with changes in the Cl^–^ coordination environment and the local catalytic site. The Cl^–^ ion remained associated with the binding site throughout all three CHARMM trajectories and two of the three Drude trajectories, with dissociation occurring at approximately 940 ns in the remaining Drude trajectory. In the enzyme–ligand simulations, the Cl^–^–Arg195 distance was similar between CHARMM (3.8 ± 0.2 Å) and Drude (3.9 ± 0.3 Å). The Cl^–^–Arg337 distance was somewhat greater in Drude (4.6 ± 0.6 Å) than in CHARMM (4.2 ± 0.3 Å). The largest difference was observed for Cl^–^–Asn298, with a substantially shorter distance in Drude (5.0 ± 0.5 Å) than in CHARMM (10 ± 2 Å), indicating a notable FF-dependent change in the local chloride coordination environment upon ligand binding. To further assess whether these differences were associated with changes in the catalytic environment, the orientation of Glu233 was evaluated using the Cα–Cβ–Cϒ–Cδ dihedral angle (Figure S22). In the enzyme–ligand simulations, CHARMM showed a dominant population near −60°, with a smaller population near ±180, whereas Drude exhibited a broader distribution with populations near approximately −100° and +80° (Figure S23). The local hydration environment of chloride was also assessed by counting water molecules within 3.5 Å of Cl^–^. The average number of waters was 1.3 ± 0.9 for CHARMM and 1.6 ± 0.7 for Drude, indicating modest differences in Cl^–^ hydration between the FFs. These results suggest that ligand binding is associated with changes in the Cl^–^ coordination environment, particularly the Cl^–^–Asn298 interaction, accompanied by differences in the conformational preferences of Glu233 and local Cl^–^ hydration.

In conclusion, the α-amylase-ligand simulations indicate that the ligand remained bound to α-amylase throughout the trajectories but adopted a dynamic and loosely defined binding mode. Unlike the MGAM systems, for which more direct interactions with catalytic residues were observed, ligand stabilization in α-amylase is likely governed by nonspecific interactions within a flexible binding environment. The Drude model may better balance these interactions but does not fundamentally alter the binding mode, highlighting the distinct nature of ligand recognition in this system.

Taken together, the three enzyme–ligand systems show that polarization can influence the balance and persistence of protein–ligand interactions, with the magnitude of these differences varying across systems. The Drude simulations generally produced more persistent and distributed interactions in the MGAM systems, whereas differences between the two FFs were less pronounced for α-amylase. The differences observed between the two FFs are consistent with the ability of the Drude model to respond to changes in the local electrostatic environment, allowing individual contacts to reorganize while maintaining ligand association with the binding site. Previous comparisons of the CHARMM and Drude models have similarly reported differences in the distribution and orientation of functional-group interactions within protein binding sites, supporting the potential influence of explicit polarization on protein–ligand interaction patterns.^38,39^ Thus, polarization does not necessarily strengthen ligand binding, but can alter the balance and persistence of individual interactions. Because the Drude model explicitly represents electronic polarization, it provides a more physically explicit treatment of environment-dependent electrostatic interactions than the fixed-charge CHARMM model; however, additional experimental or free-energy calculations would be required to determine whether the predicted binding modes are more accurate.

## Conclusions

In this study, we employed a combined FF parametrization and MD simulation approach to investigate the behavior of selected candidate inhibitors targeting key carbohydrate-digesting enzymes, including NtMGAM, CtMGAM, and pancreatic α-amylase. Ligand parameters were carefully refined using QM target data to ensure accurate representation of structural, electrostatic, and conformational properties in both the CHARMM and Drude FFs. MD simulations of the apo enzymes confirmed that the overall structural integrity of the proteins was maintained, with flexibility primarily localized to regions surrounding the catalytic pockets. These intrinsic dynamics play an important role in shaping the accessibility and geometry of the active sites. Simulations of the enzyme–ligand complexes demonstrate that the selected candidate inhibitors remained bound to their respective active sites throughout the simulations. Comparison between the CHARMM and Drude FFs revealed differences in the predicted enzyme–ligand interaction networks, with the Drude model showing more persistent and cooperative interactions. In the NtMGAM system, the ligand remained consistently bound within the catalytic pocket and formed stable hydrogen bonds with catalytic residues. In contrast, ligand binding in CtMGAM and α-amylase was characterized by greater conformational flexibility and weaker direct interactions with catalytic residues in the CHARMM simulations compared to Drude, resulting in more dynamic and less well-defined binding modes. These results demonstrate that inclusion of explicit polarization can influence the predicted interaction patterns and the ligand binding dynamics. Moreover, the stability of these binding poses and interactions with key catalytic residues provide structural support for the candidate inhibitors identified in our accompanying study on *in silico* identification of candidate inhibitors for enzymes relevant to type 2 diabetes, while experimental studies will be required to establish their binding affinity and inhibitory activity.

## Supplementary Material



## Data Availability

Small-molecule and protein coordinates and topologies are available via Zenodo at 10.5281/zenodo.20648126.

## Notes

Additional computational methods and ligand parameterization details; example ligand–water interaction geometries; ligand–water interaction energies for MAYBRIDGE-SEW03510-3 (NtMGAM), CHEMBRIDGE-57665815-3 (CtMGAM), and CHEMBRIDGE-34773317-1 (α-amylase); QM, CHARMM, and Drude dihedral potential energy surfaces for these ligands; RMSD, RMSF, and domain organization analyses of NtMGAM, CtMGAM, and α-amylase; binding-pocket volume analyses of the three enzymes; protein–ligand RMSD and ligand–catalytic-residue distance analyses; chloride coordination and Glu233 dihedral-angle analyses for α-amylase; QM/MM dipole moments and molecular polarizabilities; average binding-site volumes for NtMGAM, CtMGAM, and α-amylase (PDF)
