# Supplementary material for: Computer-Aided Drug Design for Type 2 Diabetes-Related Enzymes: Polarizable and Nonpolarizable Simulations of Enzyme-Inhibitor Complexes
Source: J Chem Inf Model. 2026 Sep 30;66(19):12955–68. doi: 10.1021/acs.jcim.6c01734 (PMC13663857; doi:10.1021/acs.jcim.6c01734)
Supplement: Supplementary file 1 [file ci6c01734_si_001.pdf]

SUPPORTING INFORMATION

Computer-Aided Drug Design for Type 2 Diabetes-Related Enzymes: Polarizable and Nonpolarizable Simulations of Enzyme-Inhibitor Complexes

Esmat Mohammadi[1,2] and Justin A. Lemkul[2,3,*]

[1] Department of Chemical Engineering, Virginia Tech, Blacksburg, VA 24061, USA
[2] Department of Biochemistry, Virginia Tech, Blacksburg, VA 24061, USA
[3] Center for Computer-Aided Drug Design, Virginia Tech, Blacksburg, VA 24061, USA

SUPPORTING FIGURES

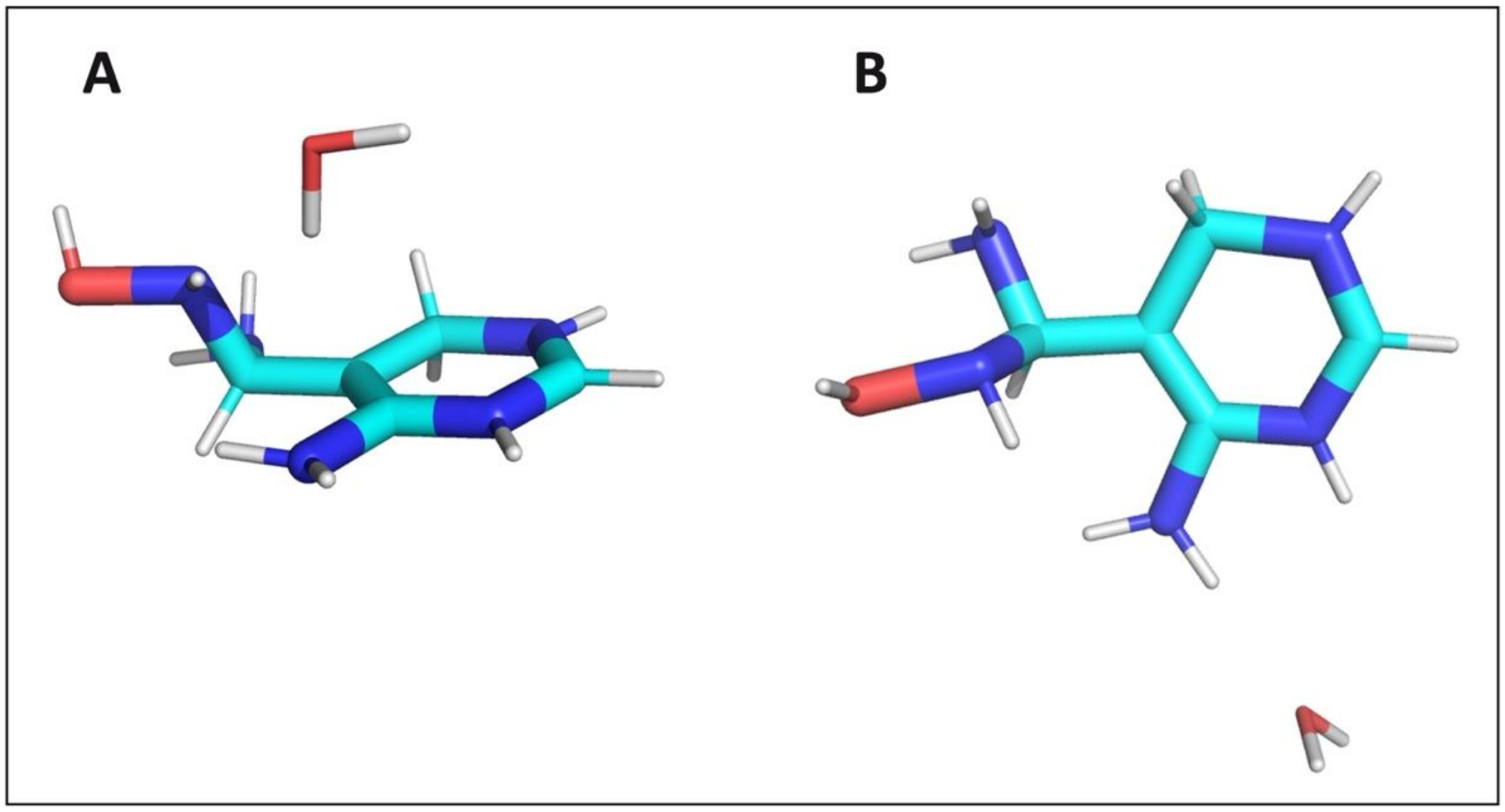

Figure S1. Illustration of example ligand–water interaction geometries used in the parametrization procedure: (A) out-of-plane (OOP) interaction and (B) 180° hydrogen-bond interaction.

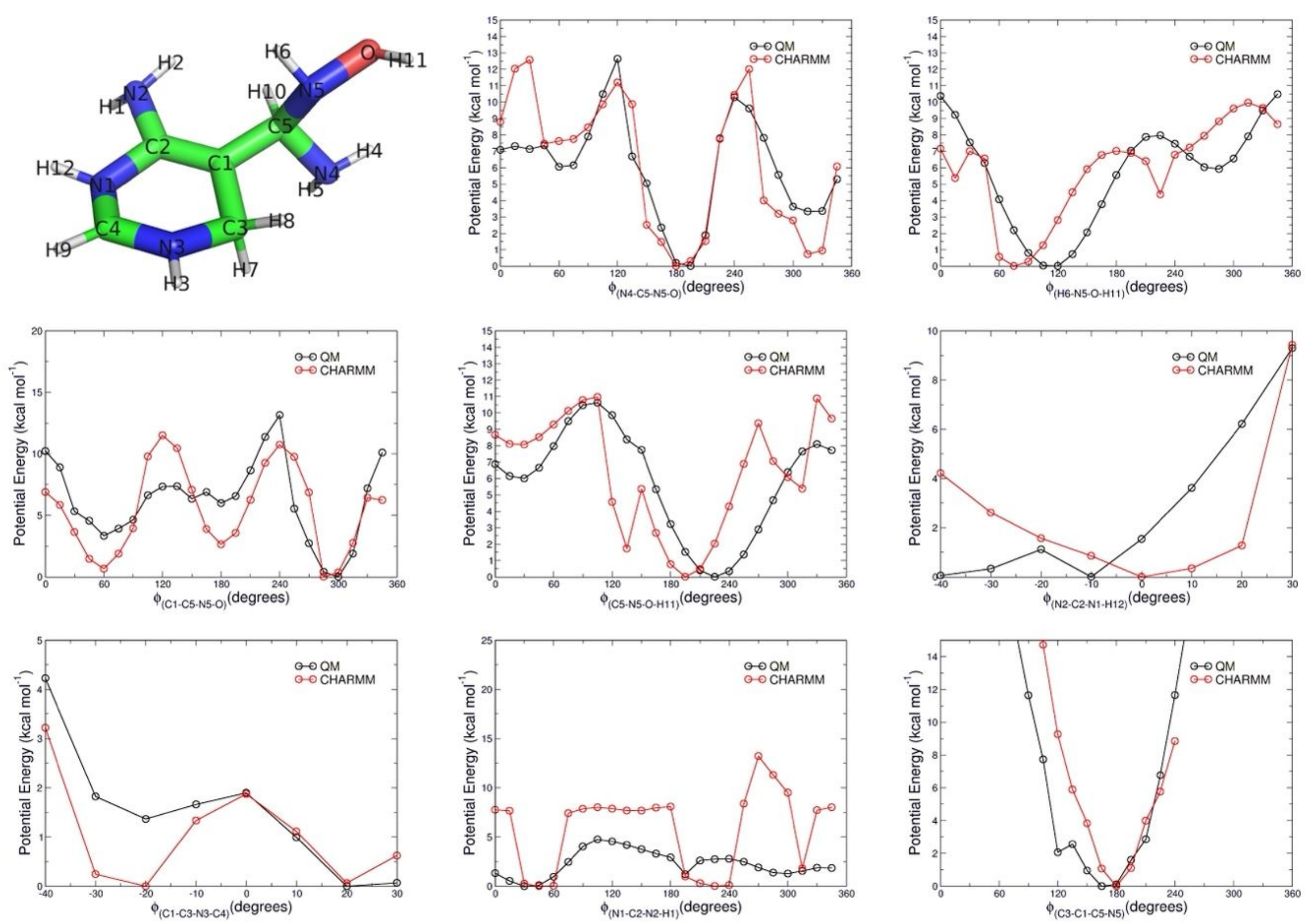

Figure S2. Comparison of QM and CHARMM dihedral potential energy surfaces for MAYBRIDGE-SEW03510-3 (NtMGAM ligand).

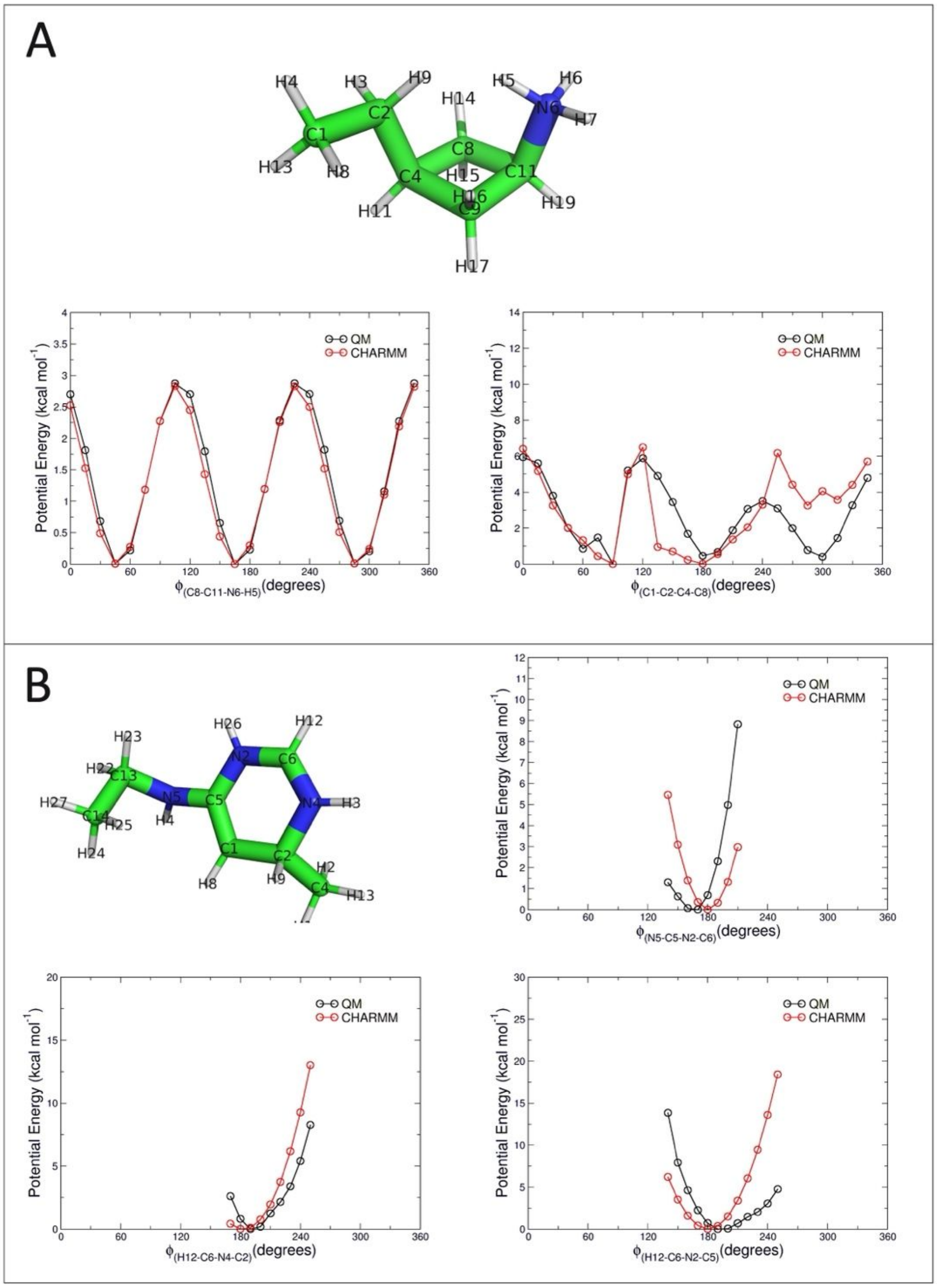

Figure S3. Comparison of QM and CHARMM dihedral potential energy surfaces for A) Section 1 and (B) Section 2 of CHEMBRIDGE-57665815-3 (CtMGAM ligand).

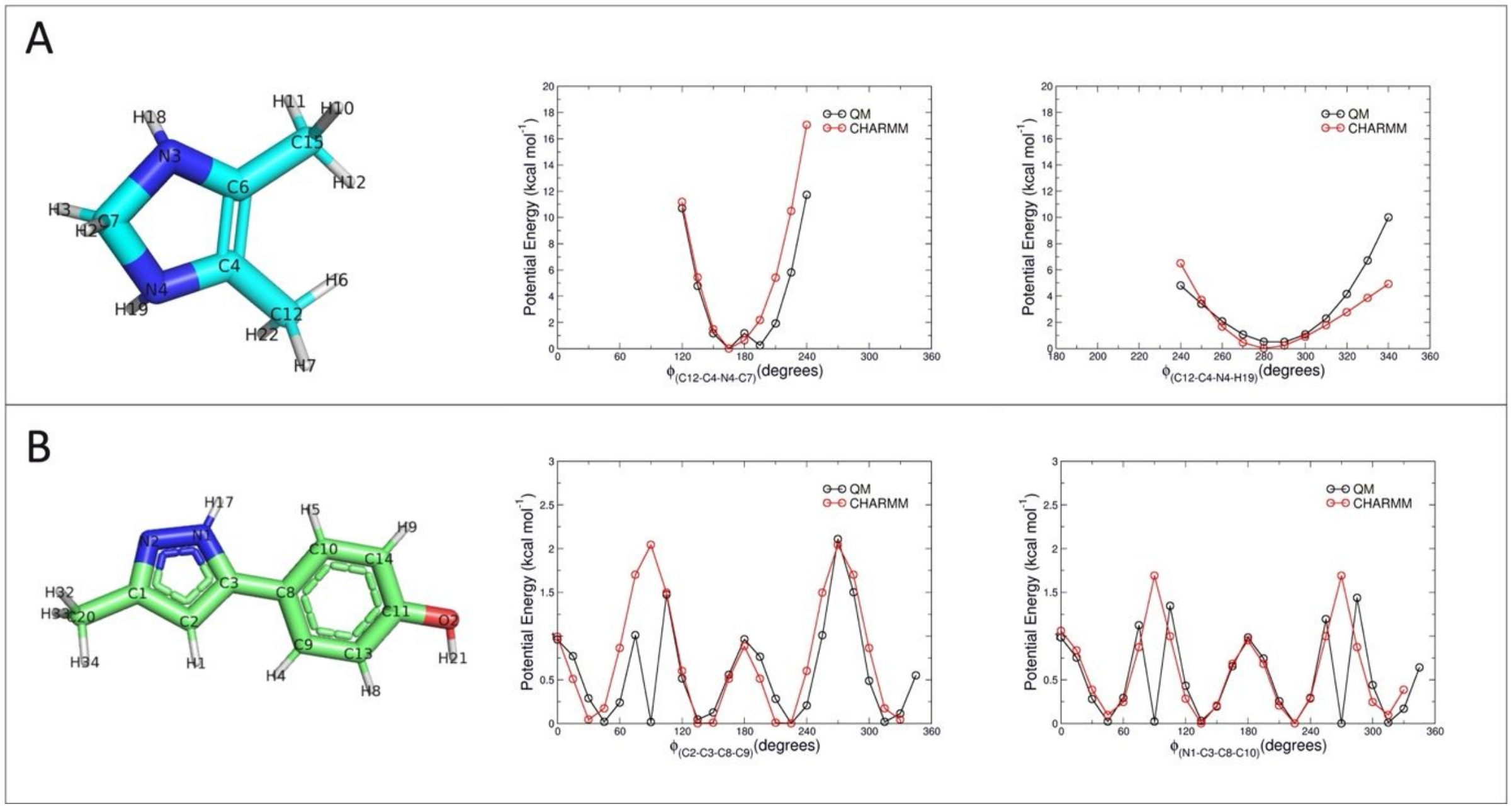

Figure S4. Comparison of QM and CHARMM dihedral potential energy surfaces for A) Section 1 and (B) Section 3 of CHEMBRIDGE-34773317-1 (α-amylase ligand).

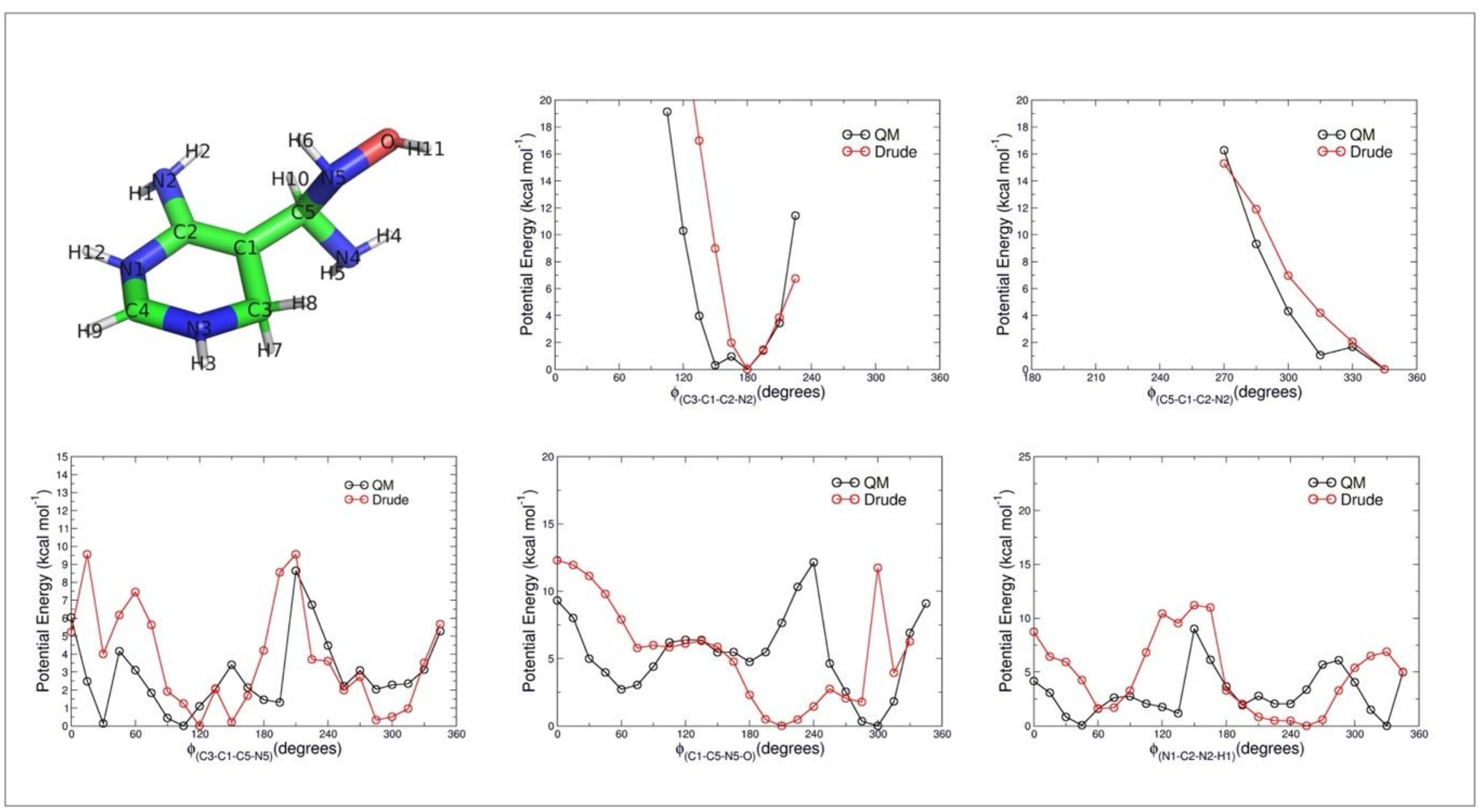

Figure S5. Comparison of QM and Drude dihedral potential energy surfaces for MAYBRIDGE-SEW03510-3 (NtMGAM ligand).

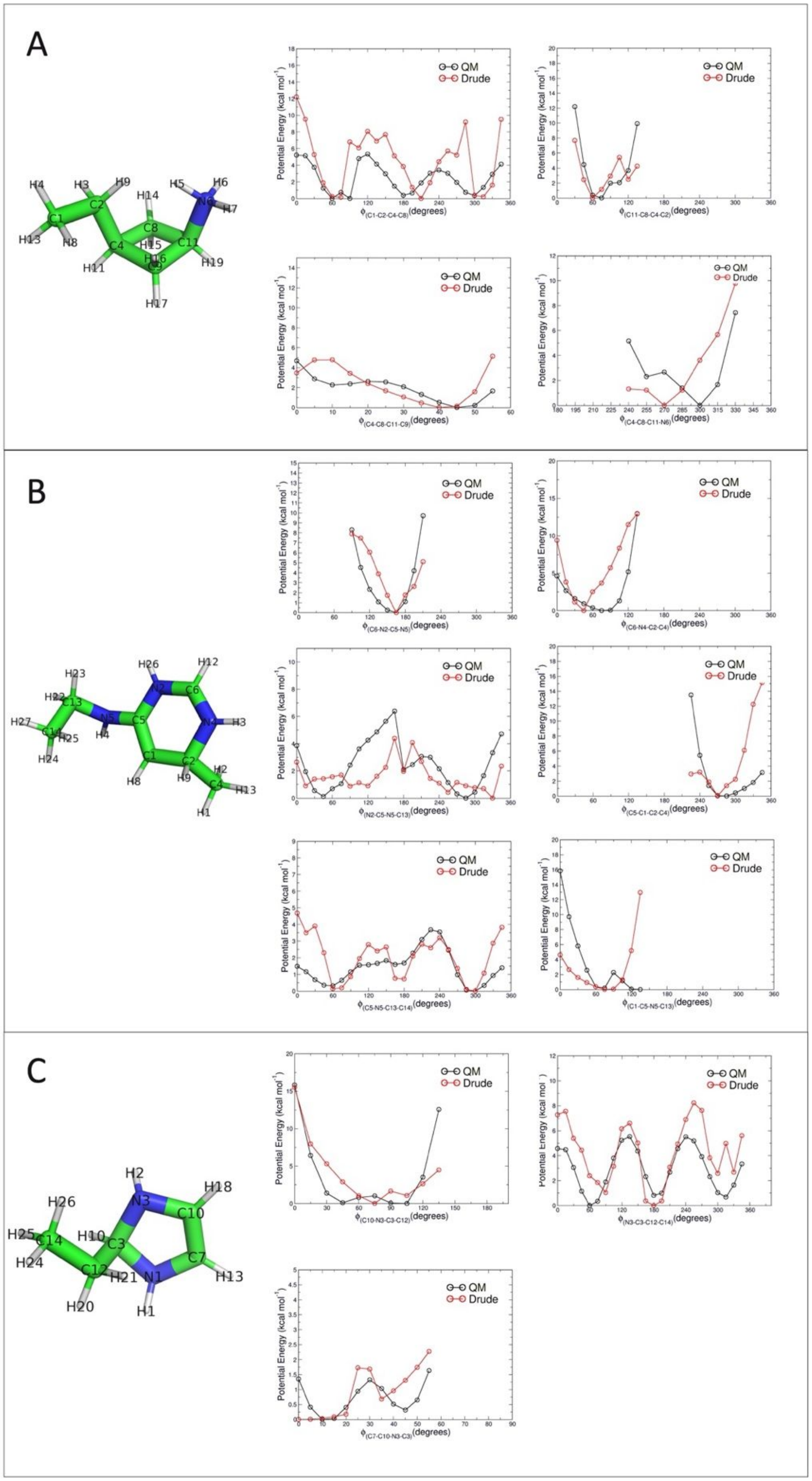

Figure S6. Comparison of QM and Drude dihedral potential energy surfaces for CHEMBRIDGE-57665815-3 (CtMGAM ligand): (A) Section 1 and (B) Section 2, and (C) Section 3.

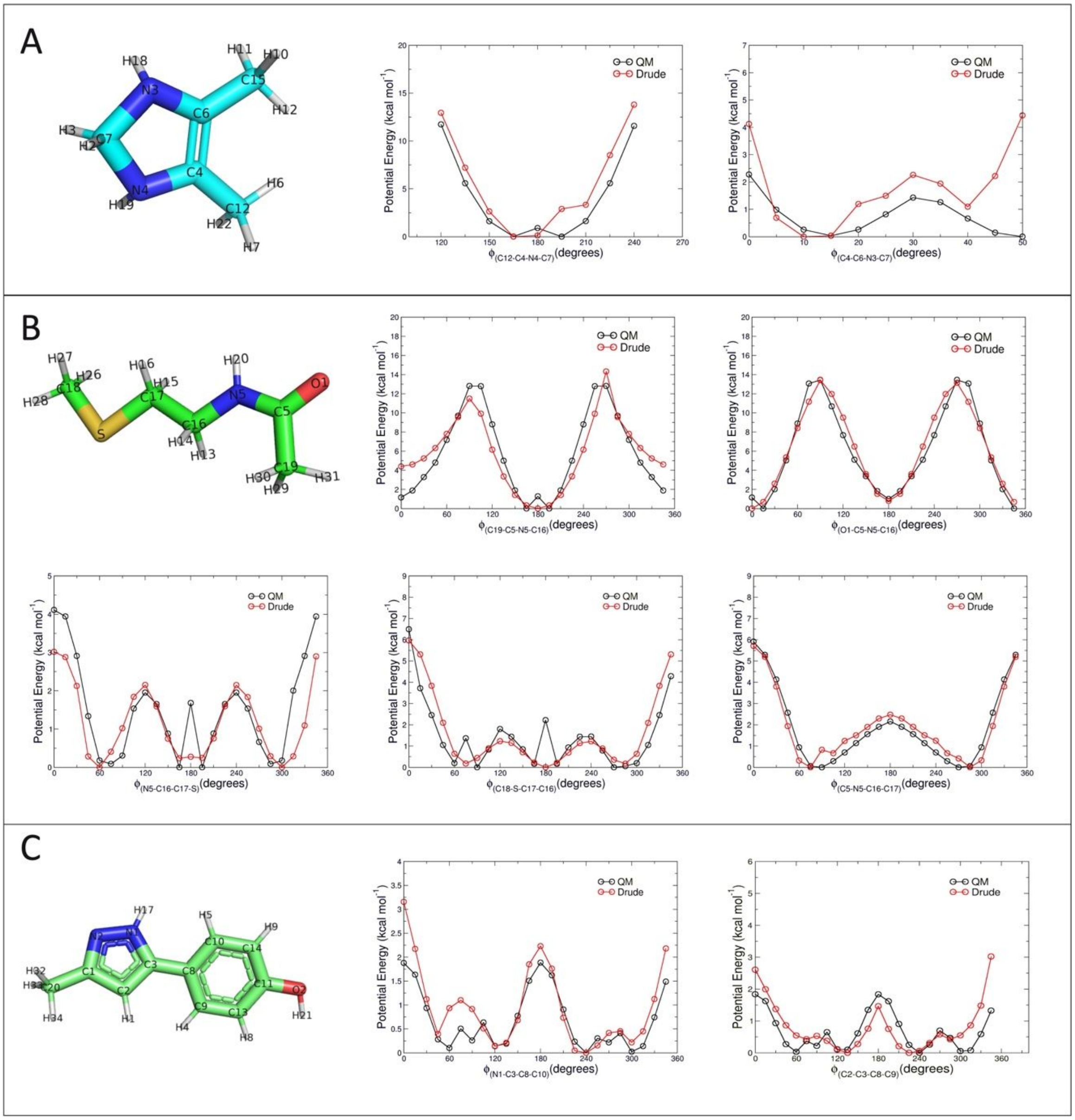

Figure S7. Comparison of QM and Drude dihedral potential energy surfaces for CHEMBRIDGE-34773317-1 (α-amylase ligand): (A) Section 1, (B) Section 2, and (C) Section 3.

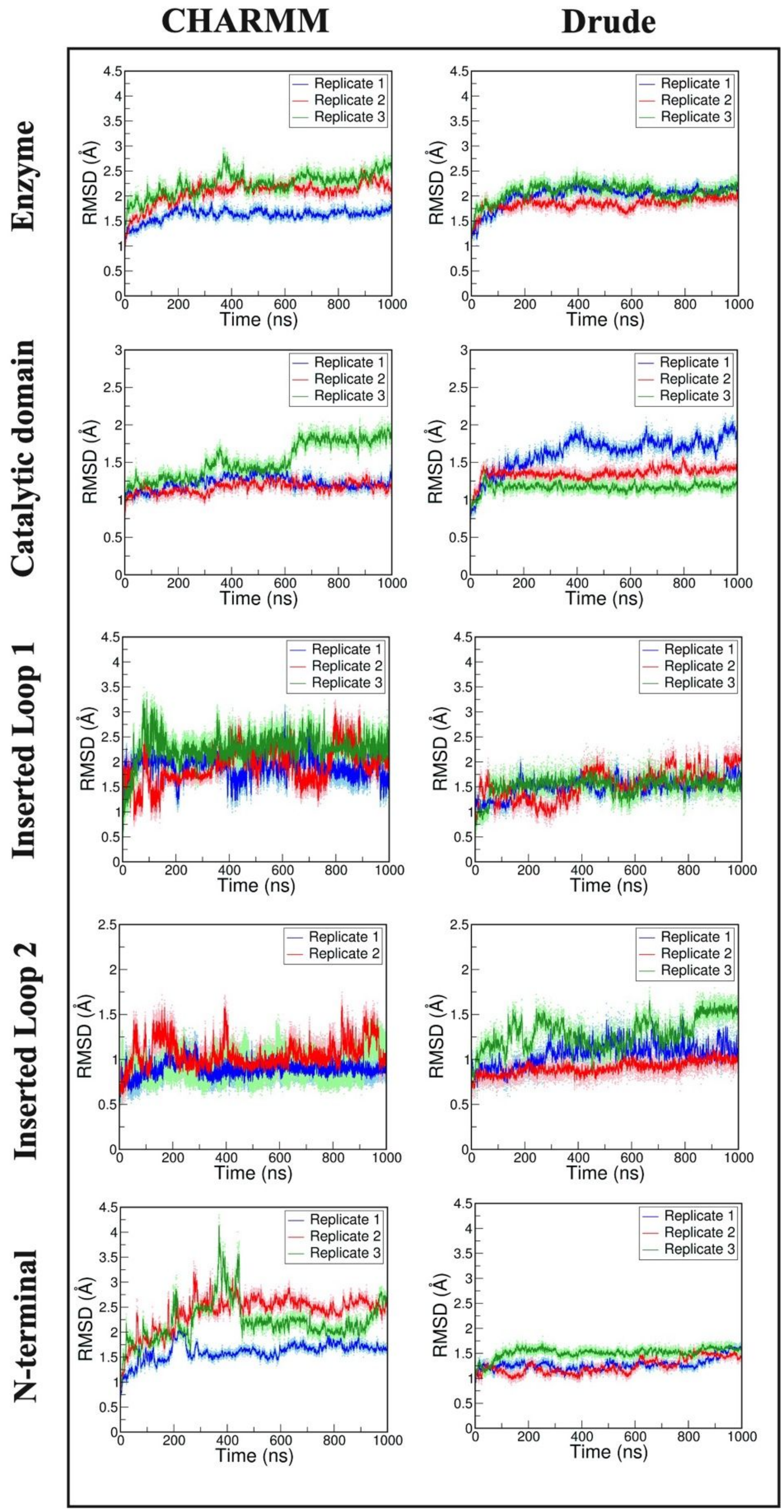

Figure S8. RMSD of Cα atoms for apo NtMGAM from three replicate simulations using the CHARMM and Drude FFs.

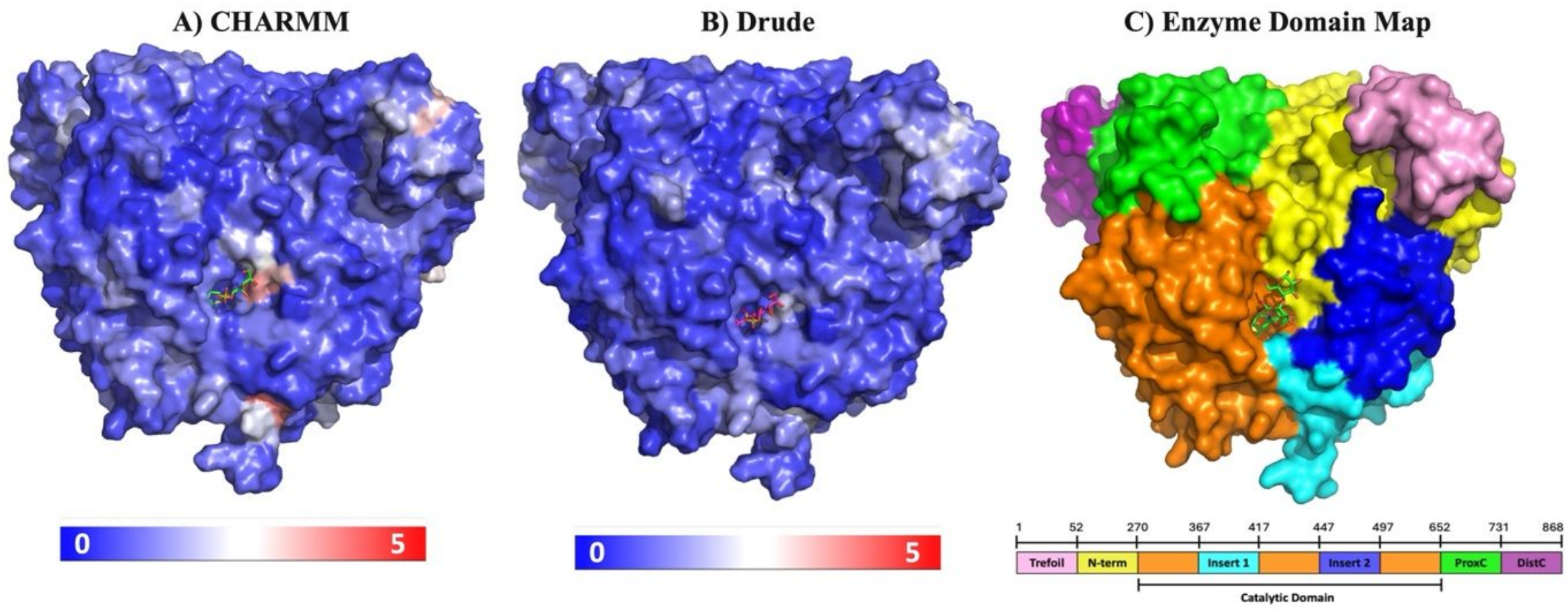

Figure S9. Surface-mapped flexibility of NtMGAM and domain organization. RMSF (Å) of Cα atoms of enzyme from MD simulations using (A) CHARMM and (B) Drude FF are projected onto the enzyme surface, illustrating the spatial distribution of residue-level flexibility (color scale: 0–5 Å). Regions of higher flexibility (red) and rigidity (blue) highlight differences in dynamic behavior between the two models. (C) Domain map of NtMGAM showing the structural organization of the enzyme.

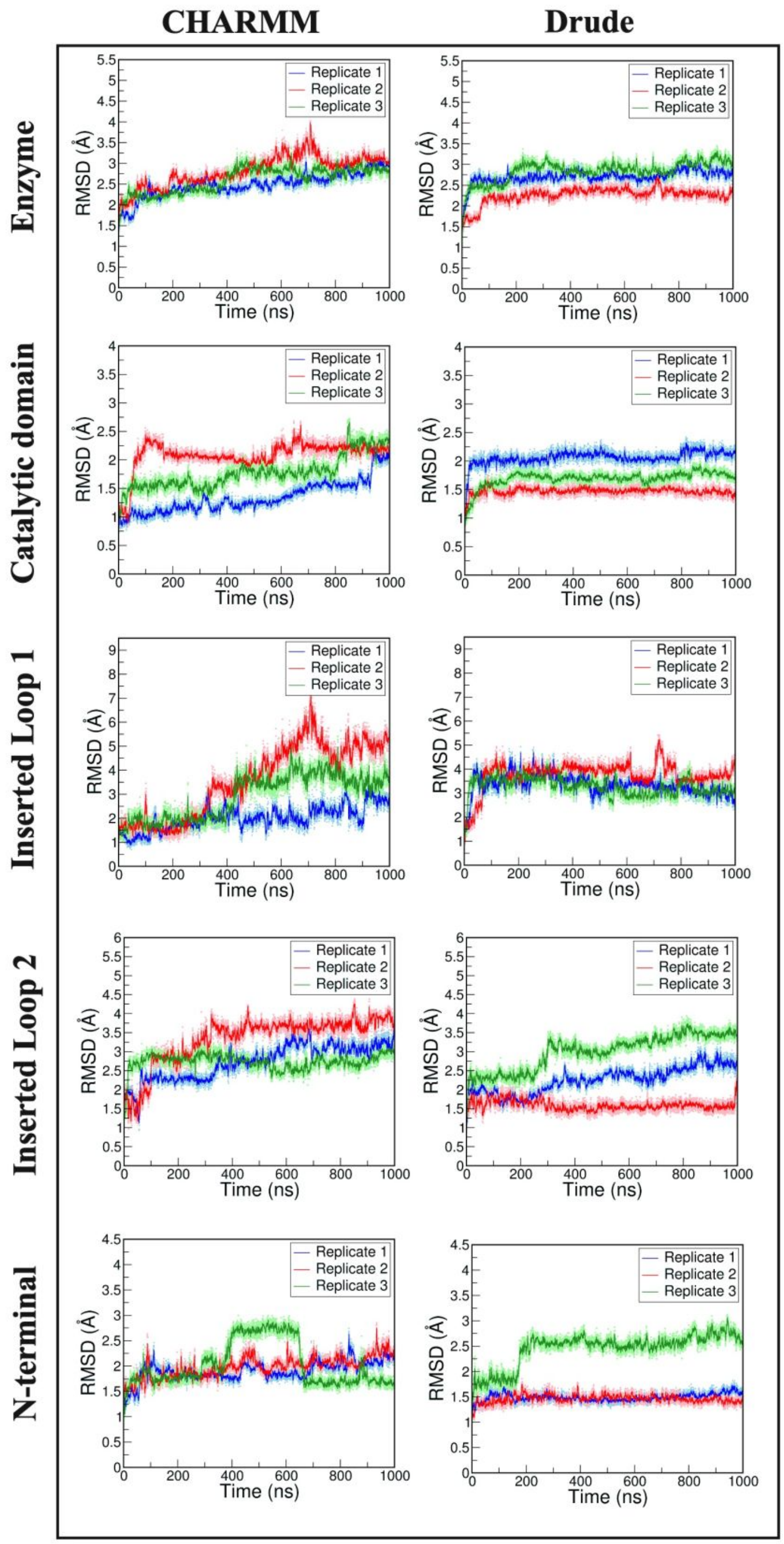

Figure S10. RMSD of Cα atoms for apo CtMGAM from three replicate simulations using the CHARMM and Drude FFs.

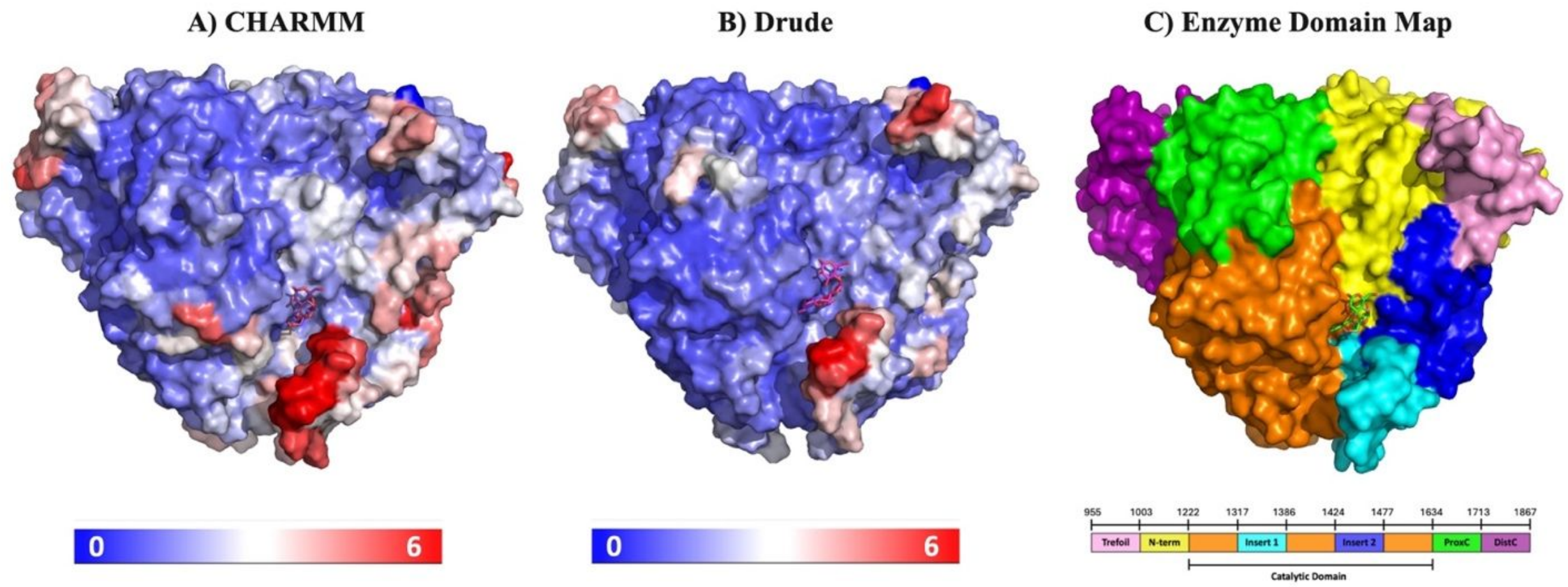

Figure S11. Surface-mapped flexibility of CtMGAM and domain organization. RMSF (Å) of Cα atoms of enzyme from MD simulations using (A) CHARMM and (B) Drude FF are projected onto the enzyme surface, illustrating the spatial distribution of residue-level flexibility (color scale: 0–6 Å). Regions of higher flexibility (red) and rigidity (blue) highlight differences in dynamic behavior between the two models. (C) Domain map of CtMGAM showing the structural organization of the enzyme.

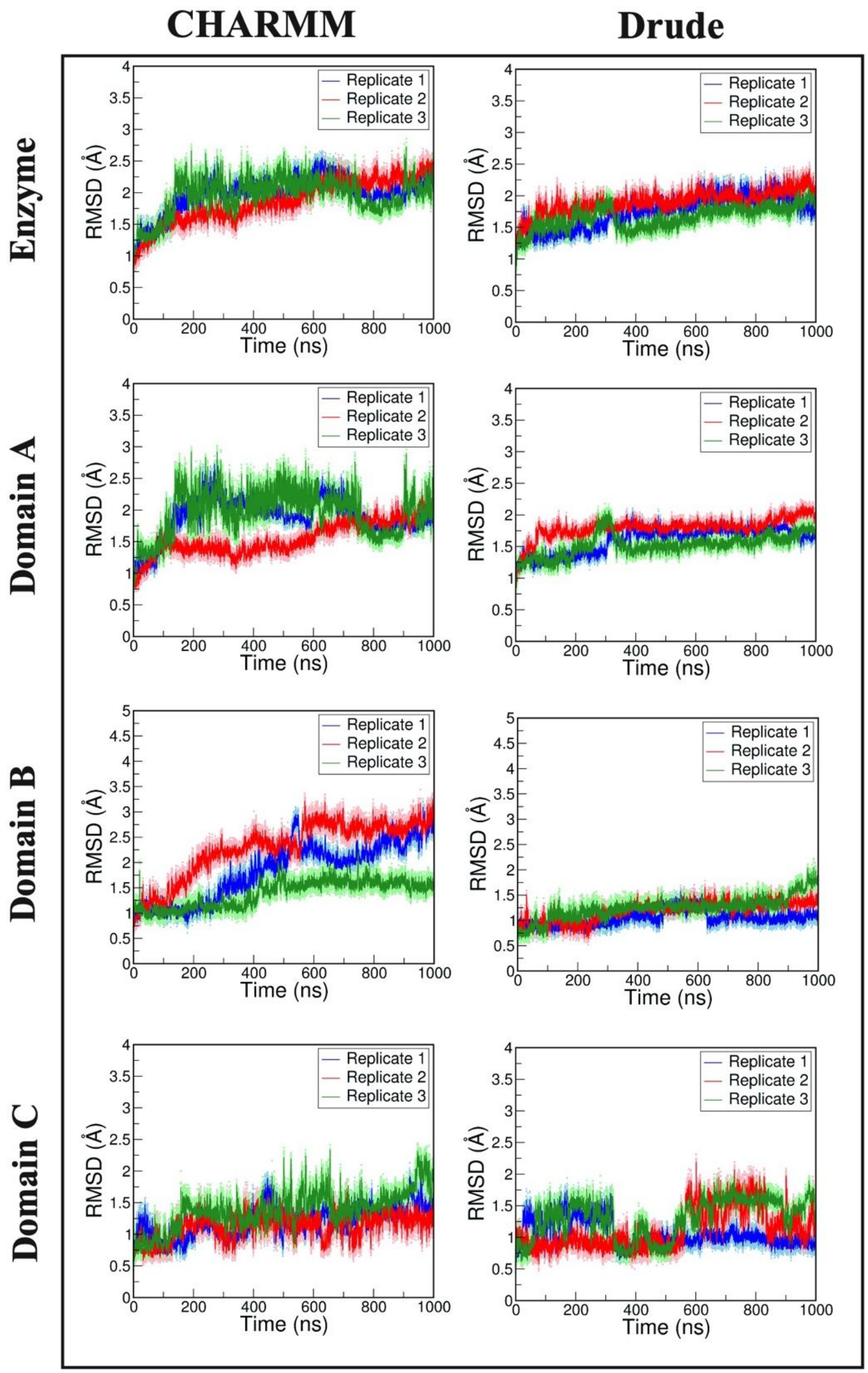

Figure S12. RMSD of Cα atoms for apo α-amylase calculated for Domains A, B, and C from three replicate simulations using the CHARMM and Drude FFs.

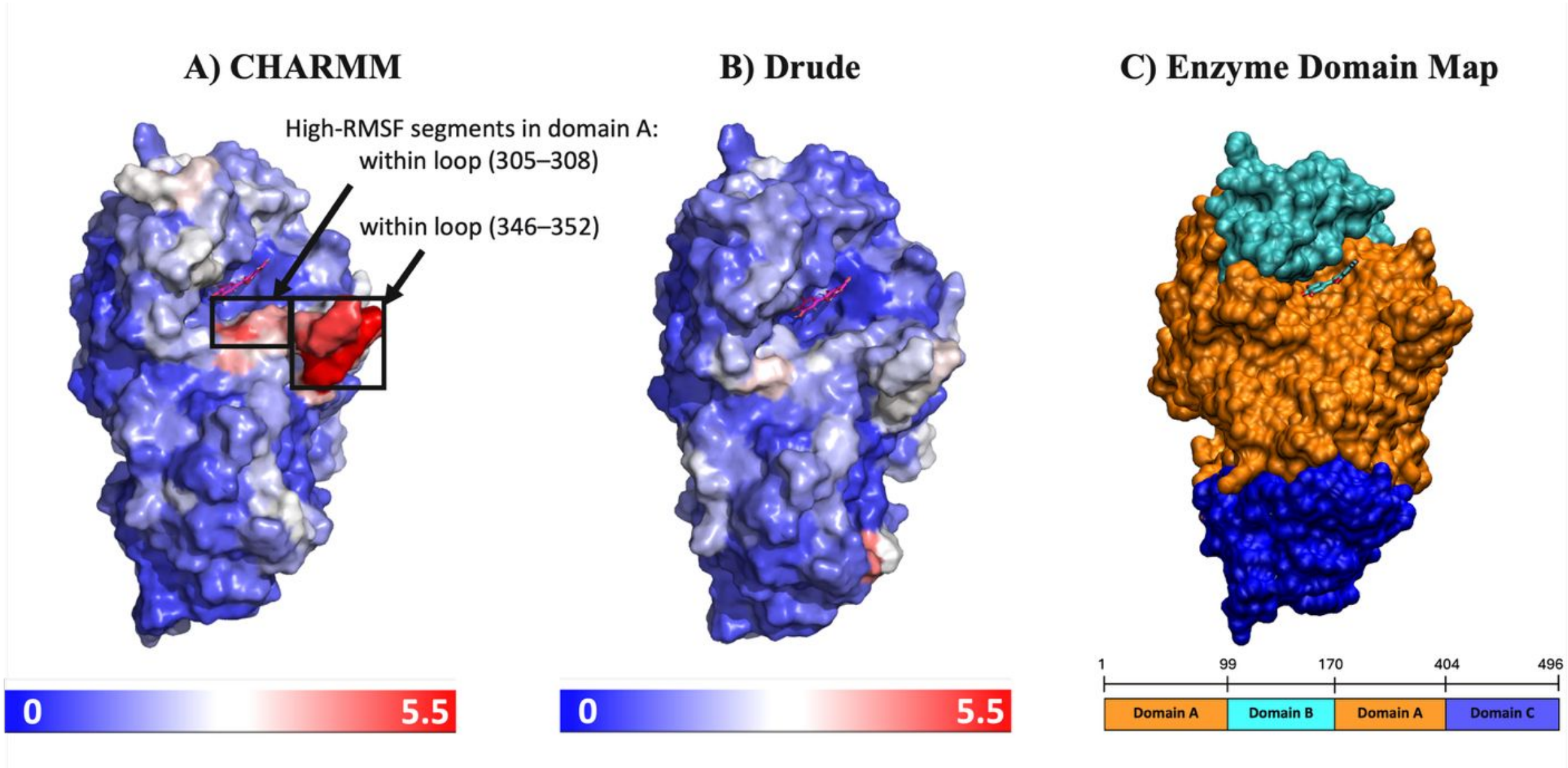

Figure S13. RMSF (Å) of Cα atoms mapped onto the surface of α-amylase. Surface-mapped flexibility of α-amylase and domain organization. RMSF (Å) of Cα atoms of enzyme from MD simulations using (A) CHARMM (The prominent high-RMSF segments at residues 305–308 and 346–352 within Domain A are indicated) and (B) Drude FF are projected onto the enzyme surface, illustrating the spatial distribution of residue-level flexibility (color scale: 0–5.5 Å). Regions of higher flexibility (red) and rigidity (blue) highlight differences in dynamic behavior between the two models. (C) Domain architecture of α-amylase, showing the structural segmentation into Domains A–C.

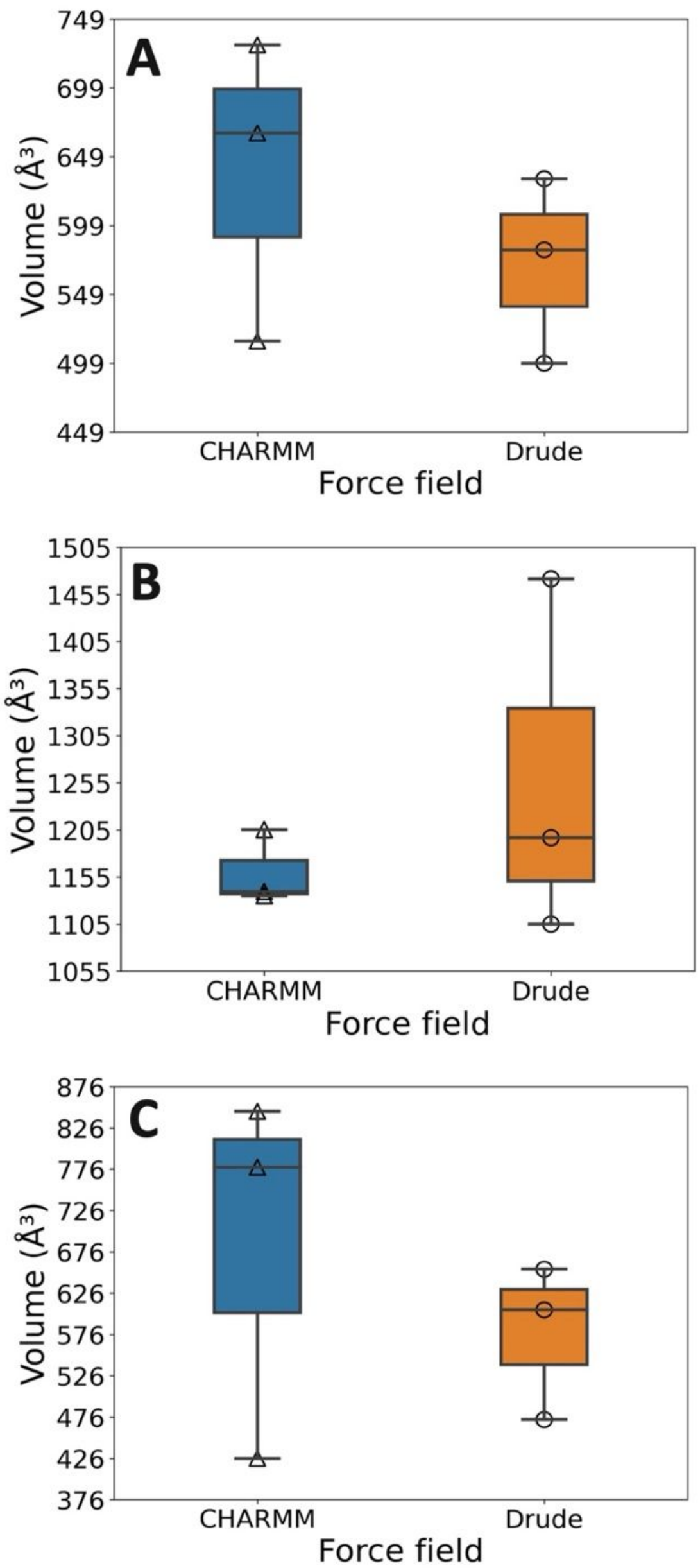

Figure S14. Box plots illustrating the distribution of pocket volumes sampled during the three replicate simulations for (A) NtMGAM, (B) CtMGAM, and (C) α-amylase. The plots highlight the variability of pocket volumes across trajectories. The boxes represent the interquartile range (25th–75th percentiles), the central line indicates the median, and whiskers extend to the minimum and maximum values.

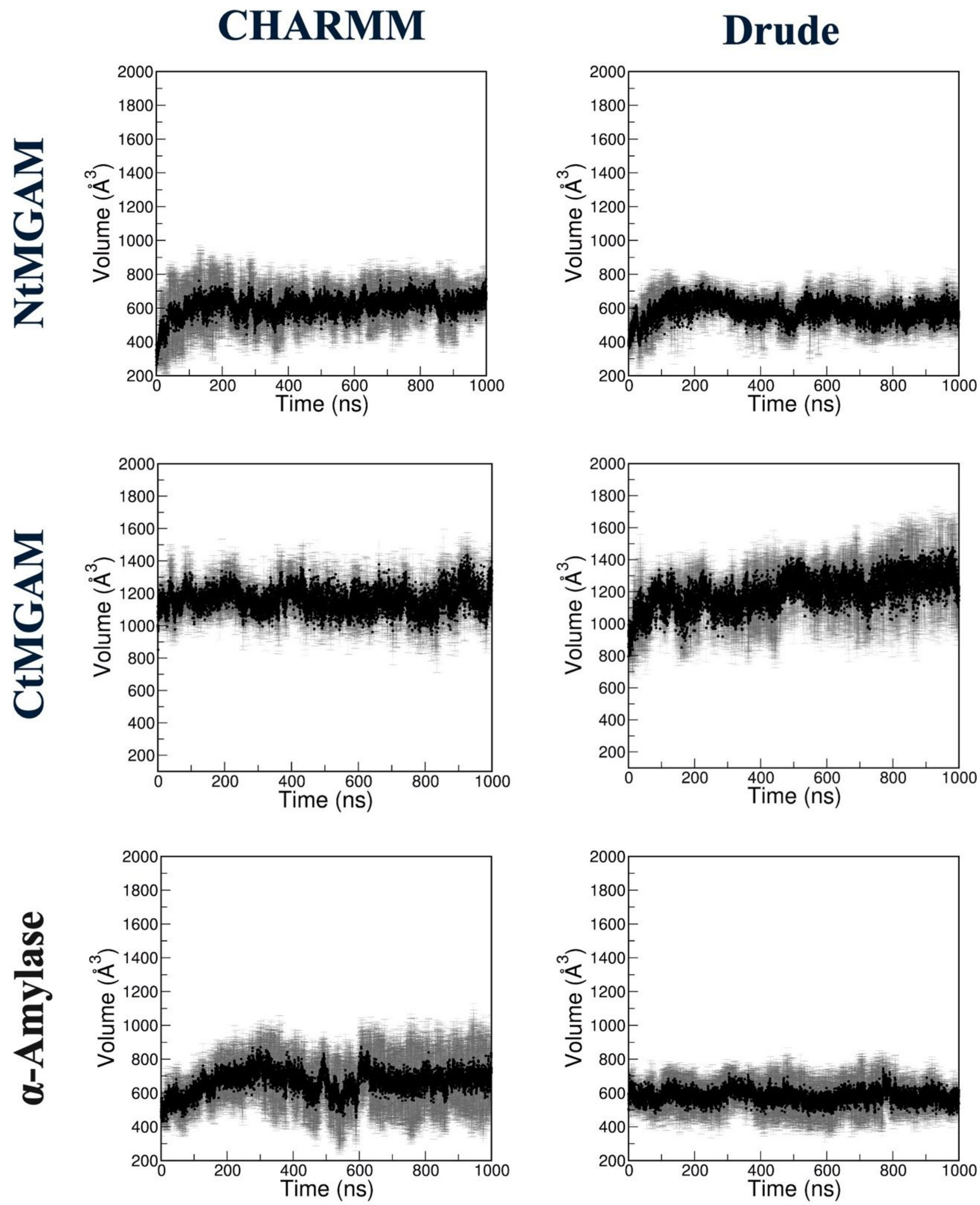

Figure S15. Time evolution of binding pocket volume for NtMGAM, CtMGAM, and α-amylase from CHARMM and Drude simulations. Values are averaged over three replicate simulations, with standard deviations at each time point shown as error bars.

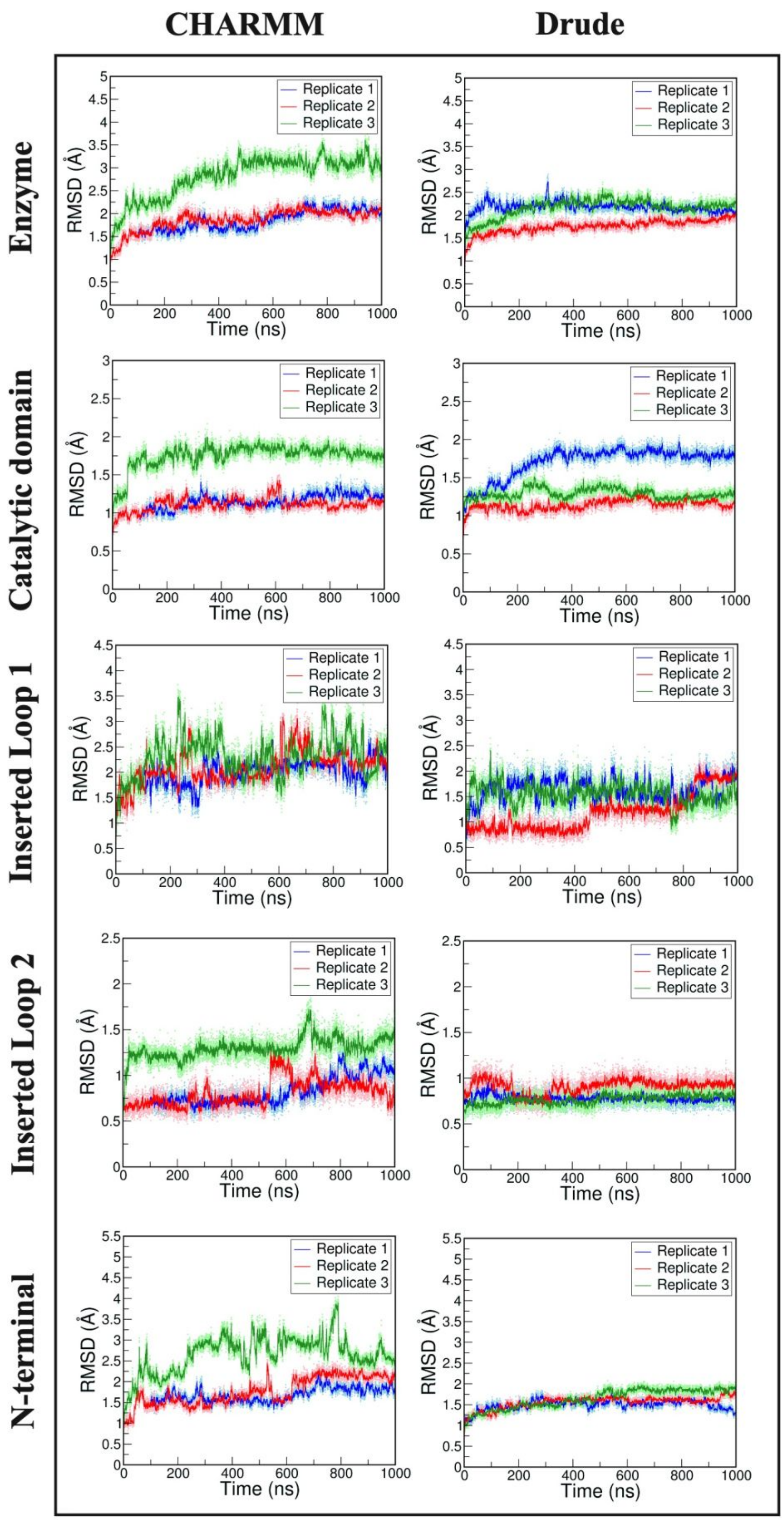

Figure S16. RMSD of Cα atoms for NtMGAM-ligand complex calculated for three replicate simulations using the CHARMM and Drude FFs.

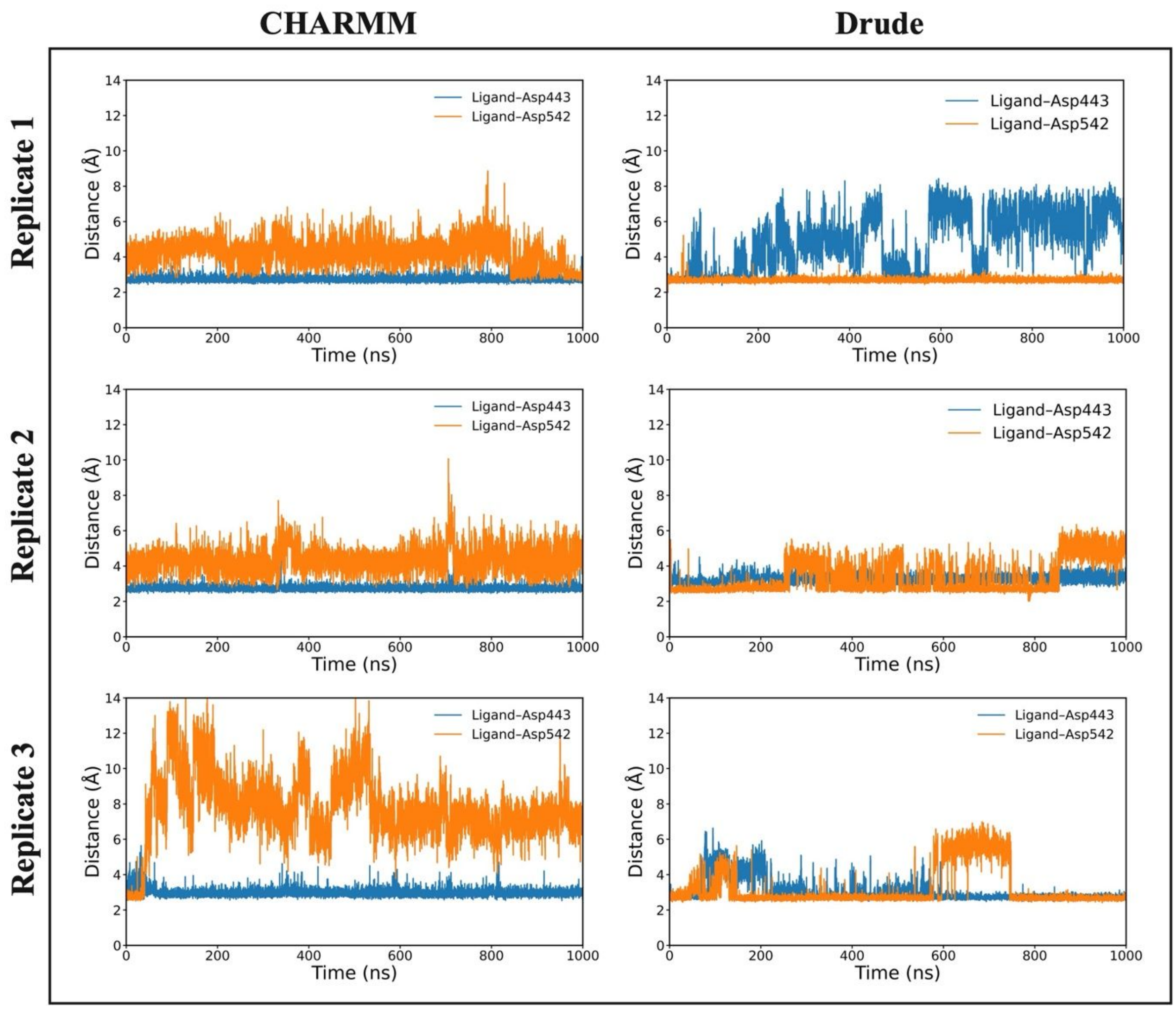

Figure S17. Minimum heavy-atom distance between the ligand and the catalytic carboxylate oxygen atoms in NtMGAM, calculated as the shortest pairwise distance at each simulation frame for CHARMM and Drude FFs.

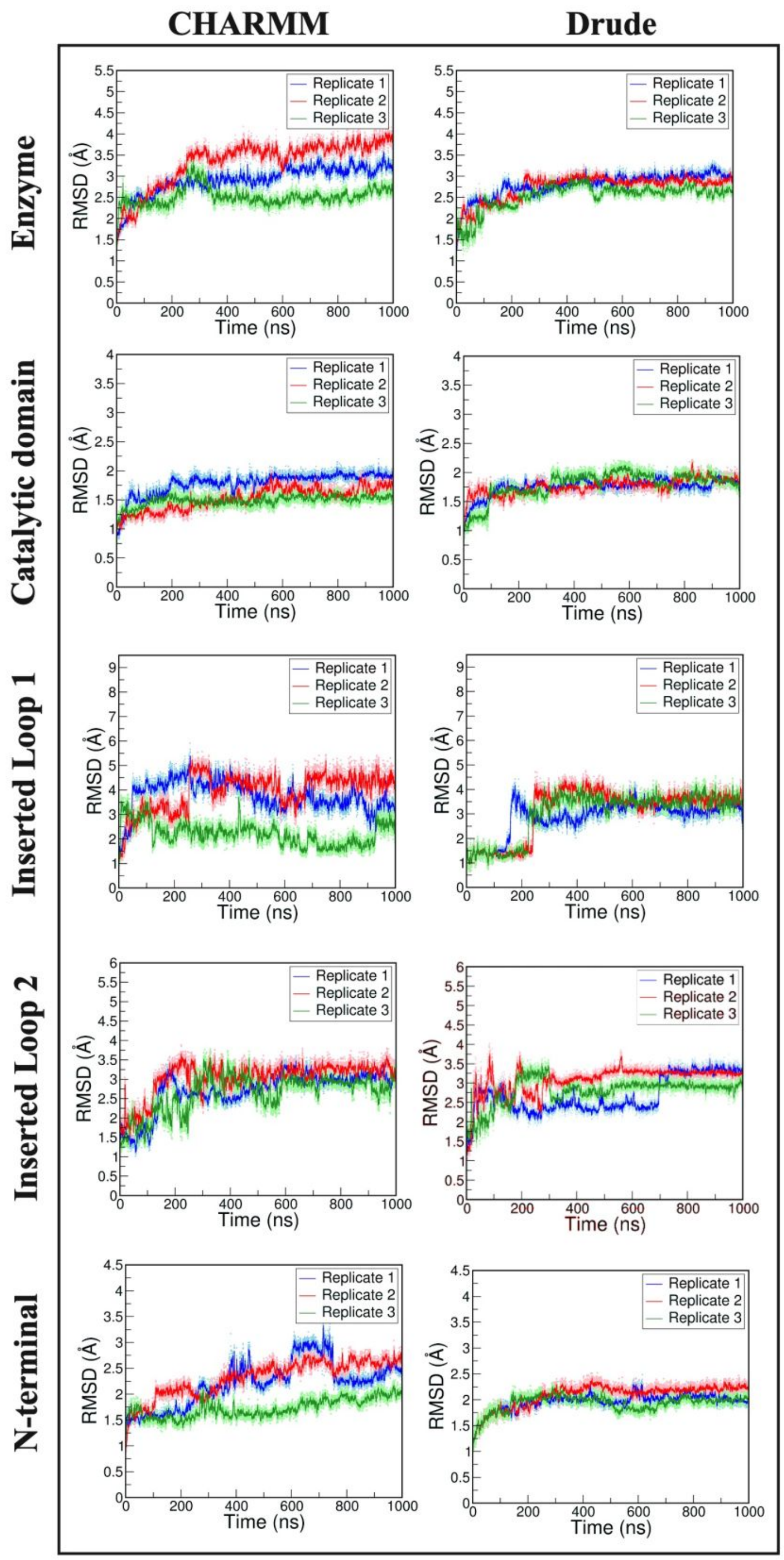

Figure S18. RMSD of Cα atoms for CtMGAM-ligand complex calculated for three replicate simulations using the CHARMM and Drude FFs.

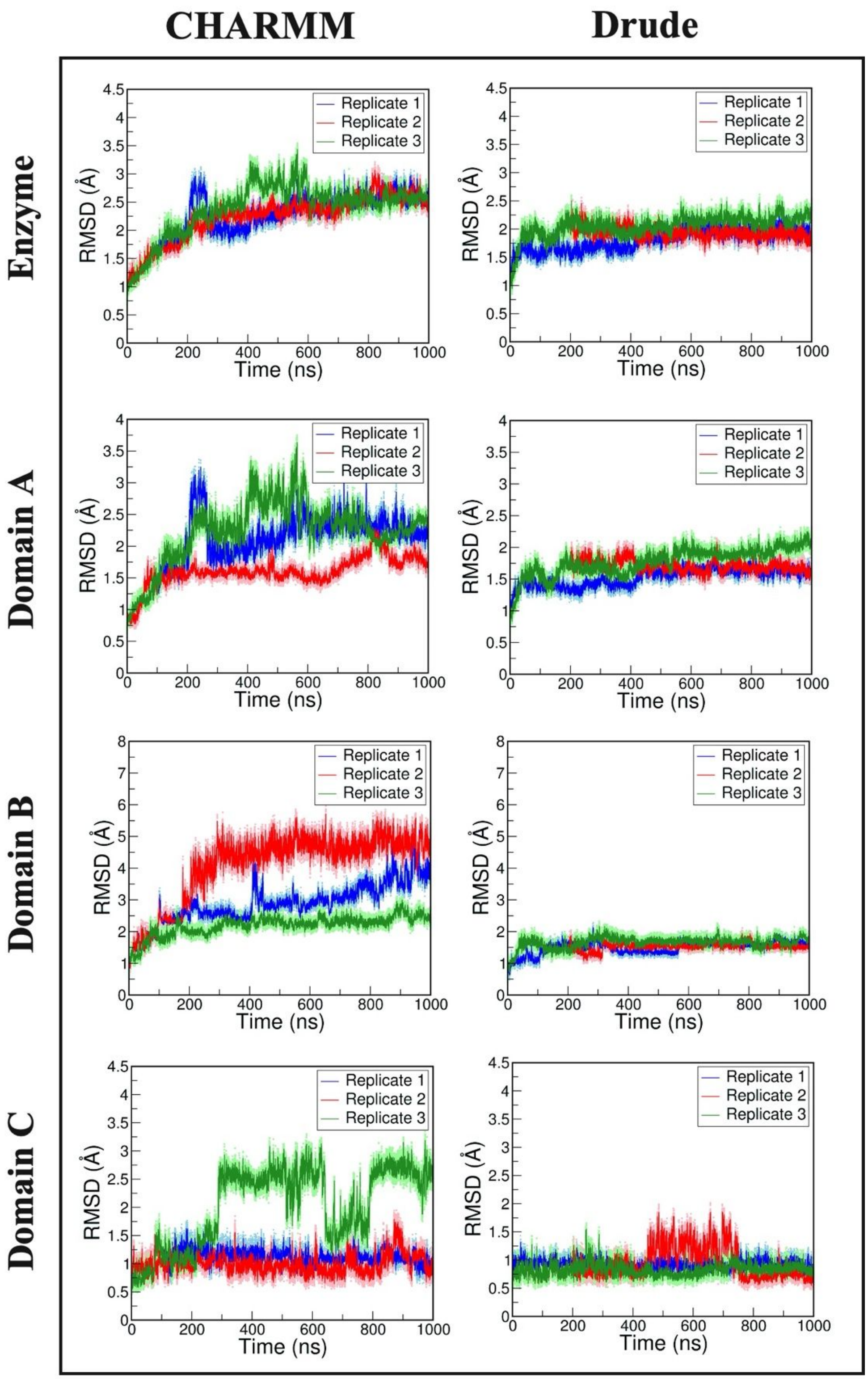

Figure S19. RMSD of Cα atoms for α-amylase-ligand complex calculated for three replicate simulations using the CHARMM and Drude FFs.

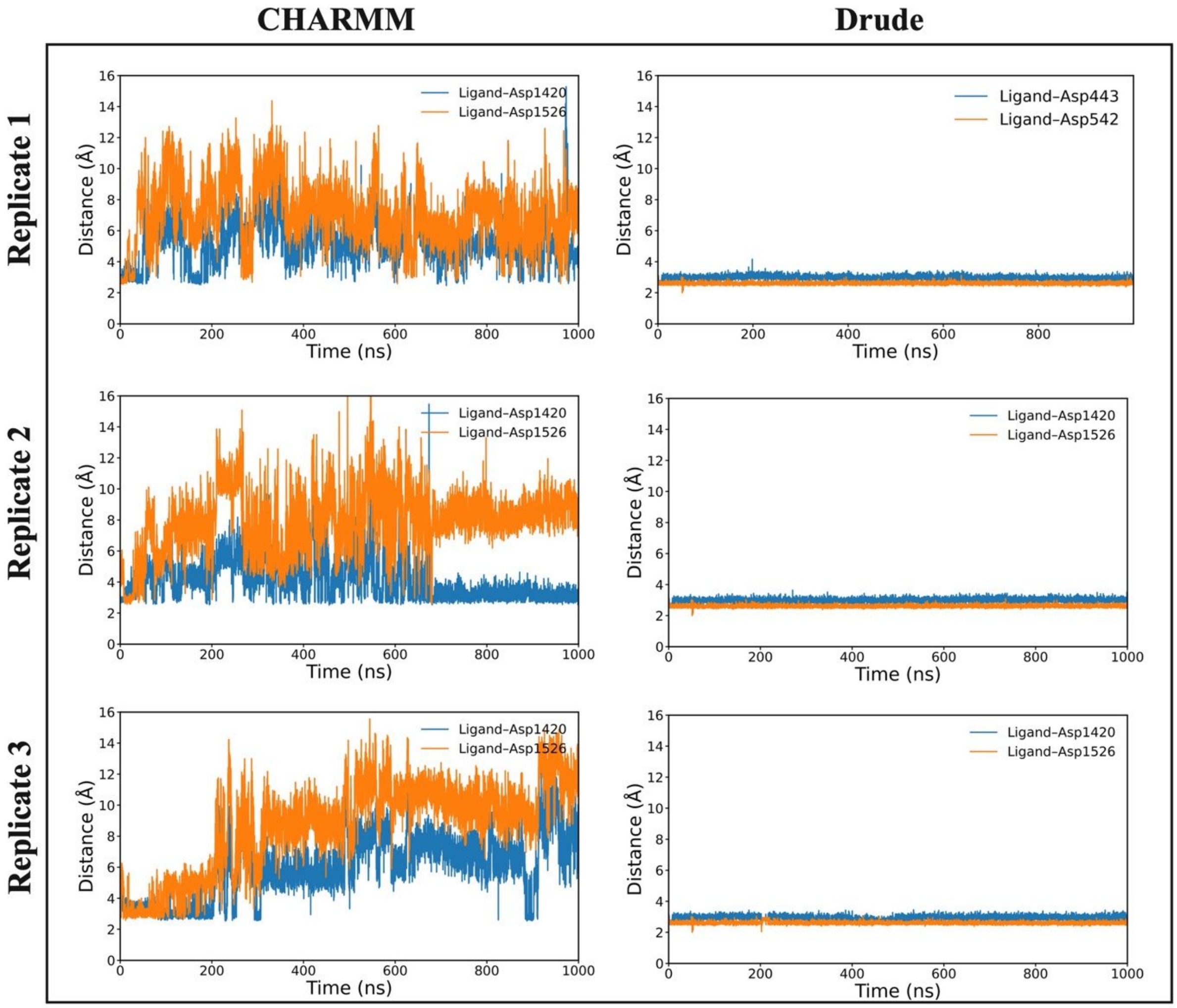

Figure S20. Minimum heavy-atom distance between the ligand and the catalytic carboxylate oxygen atoms in CtMGAM, calculated as the shortest pairwise distance at each simulation frame for CHARMM and Drude FFs.

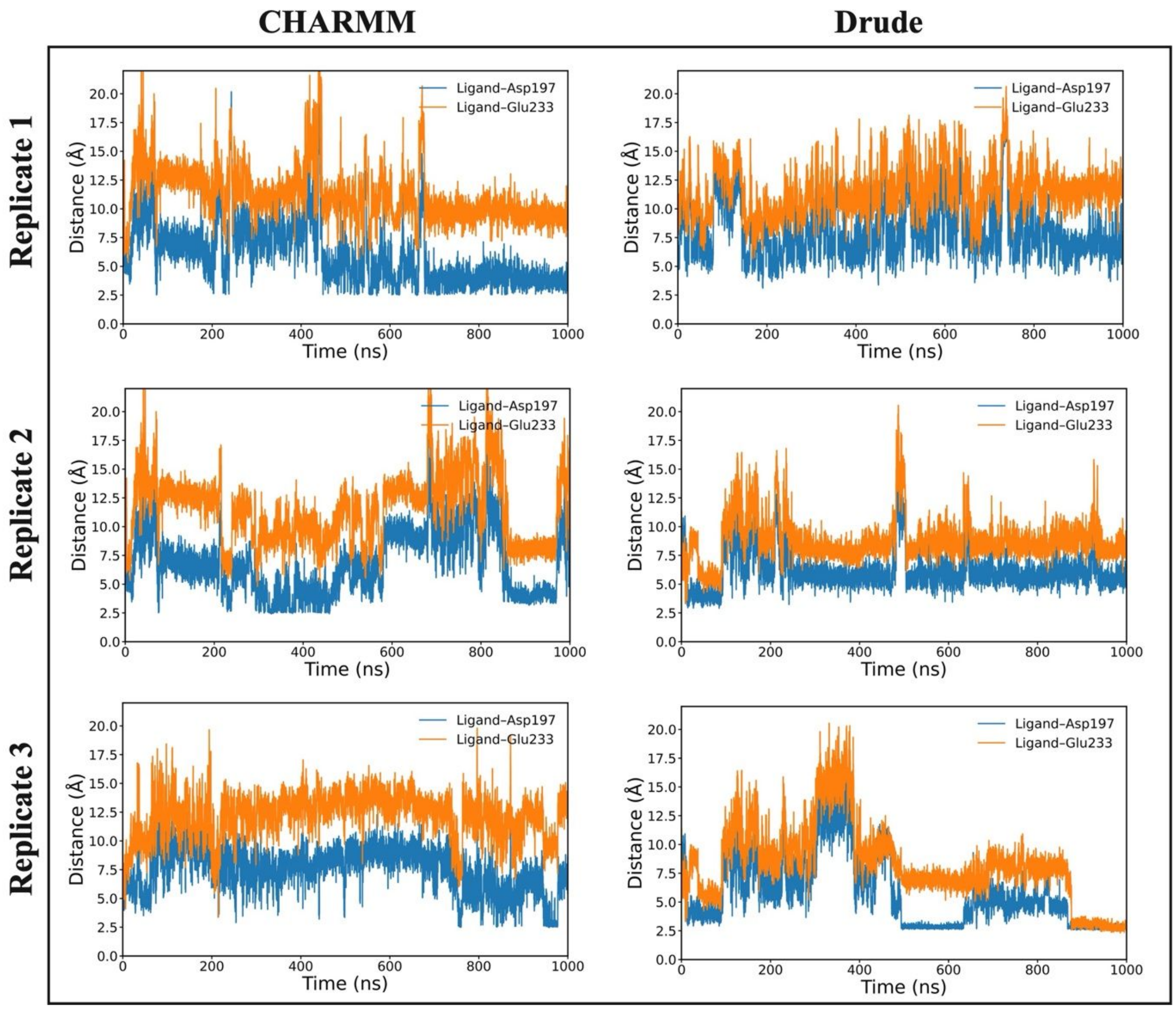

Figure S21. Minimum heavy-atom distance between the ligand and the catalytic carboxylate oxygen atoms in α-amylase, calculated as the shortest pairwise distance at each simulation frame for CHARMM and Drude FFs.

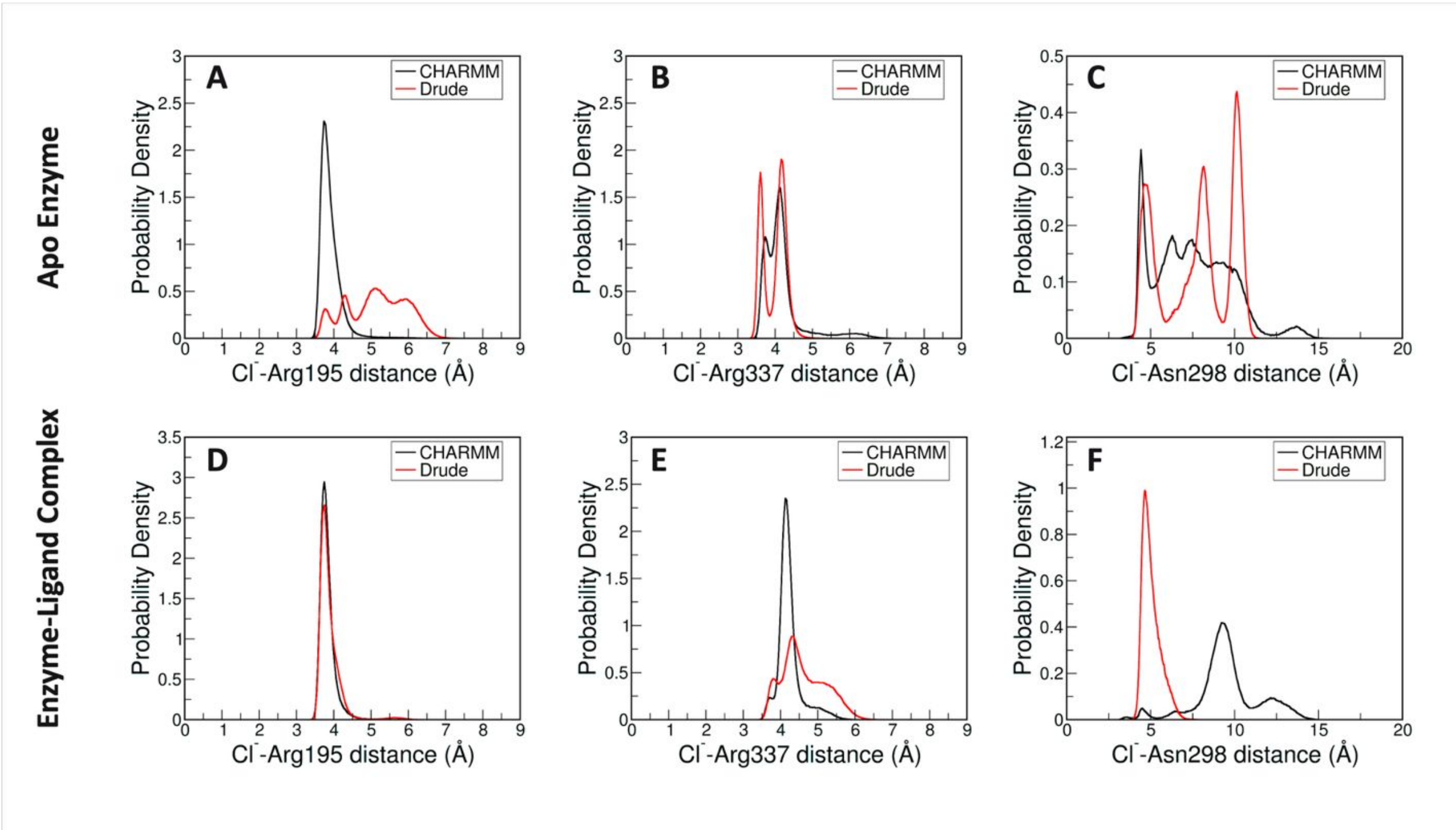

Figure S22. Probability density distributions of the distances between Cl^- and the selected chloride-coordinating residues in pancreatic α-amylase for the CHARMM (black) and Drude (red) force fields. (A–C) Apo enzyme: (A) Cl^-–Arg195, (B) Cl^-–Arg337, and (C) Cl^-–Asn298 distances. (D–F) Enzyme–ligand complex: (D) Cl^-–Arg195, (E) Cl^-–Arg337, and (F) Cl^-–Asn298 distances.

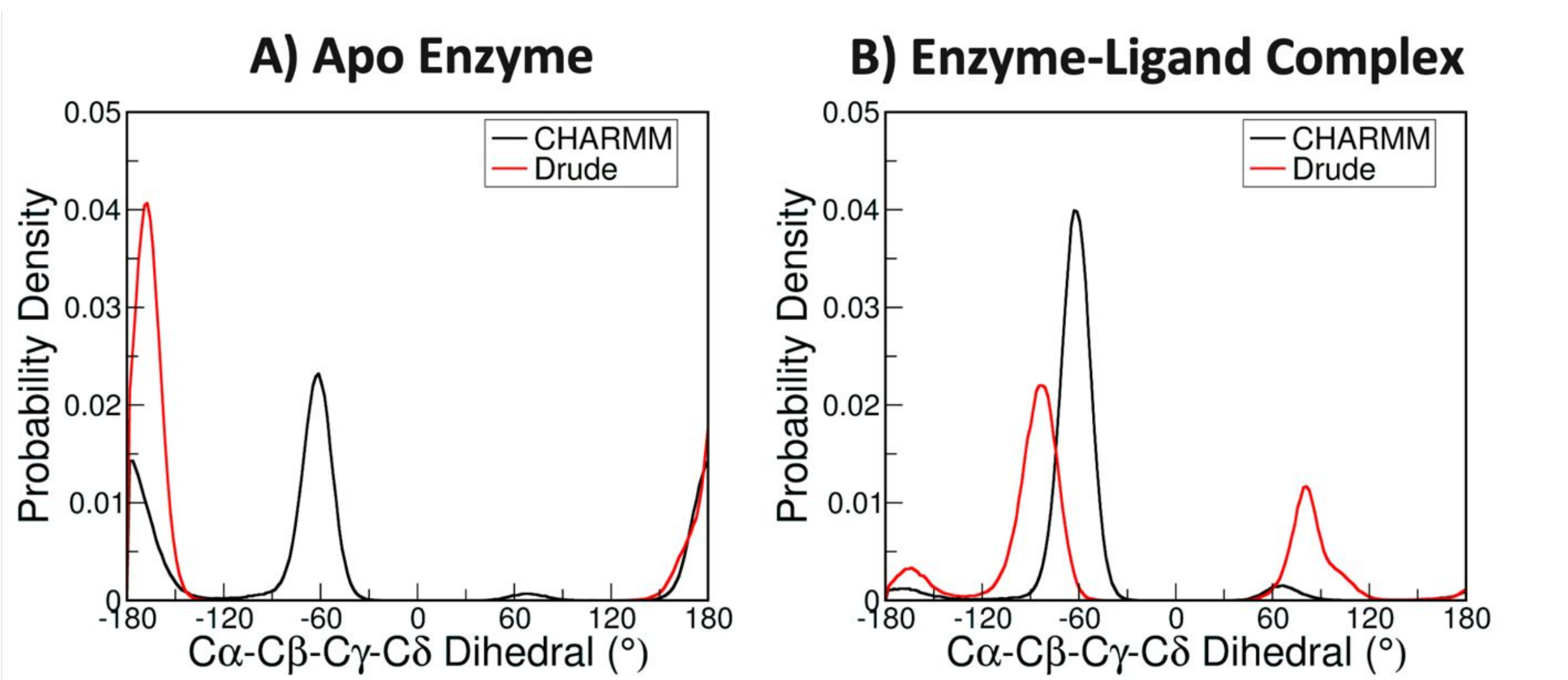

Figure **S23**. Probability density distributions of the Cα–Cβ–Cγ–Cδ dihedral angle of Glu233 in pancreatic α-amylase simulations using the CHARMM (black) and Drude (red) force fields. (A) Apo enzyme and (B) enzyme–ligand complex.

SUPPORTING TABLES

Table S1. Comparison of QM and MM dipole moment components for the three ligands using the CHARMM FF. QM dipole moment components are scaled by a factor of 1.15. Subsection 2 of CHEMBRIDGE-34773317-1 exhibited very low CGenFF penalty values and therefore did not require reparametrization, which is reflected by the absence of dipole moment values.

Ligand	Subsections	QM Dipole Moment (D)				MM Dipole Moment (D)			
		μ_x	μ_y	μ_z	$\|\mu\|$	μ_x	μ_y	μ_z	$\|\mu\|$
MAYBRIDGE-SEW03510-3	Section 1	5.33	5.59	0.54	7.74	4.45	5.82	1.65	7.51
CHEMBRIDGE-57665815-3	Section 1	13.21	2.92	3.92	14.09	12.69	3.56	-1.12	13.22
	Section 2	3.74	6.61	5.45	9.35	4.95	8.01	2.95	9.86
	Section 3	-0.65	-0.66	0.26	0.96	-0.64	-0.04	0.44	0.78
CHEMBRIDGE-34773317-1	Section 1	-0.31	0.42	-2.29	2.35	-0.49	0.66	-2.23	2.37
	Section 2	-	-	-	-	-	-	-	-
	Section 3	1.49	2.58	0.88	3.11	0.65	2.49	0.56	2.63

Table S2. Comparison of QM and MM dipole moment values for the three ligands using the Drude polarizable FF.

Ligand	Subsections	QM Dipole Moment (D)				MM Dipole Moment (D)			
		μ_x	μ_y	μ_z	$\|\mu\|$	μ_x	μ_y	μ_z	$\|\mu\|$
MAYBRIDGE-SEW03510-3	Section 1	4.63	4.86	0.61	6.74	5.97	3.71	-1.07	7.11
CHEMBRIDGE-57665815-3	Section 1	11.32	2.53	-3.35	12.07	10.68	3.28	-2.46	11.44
	Section 2	3.13	5.64	4.85	8.07	2.97	5.17	5.50	8.11
	Section 3	-0.62	-0.50	0.27	0.84	-0.53	-0.68	-0.12	0.87
CHEMBRIDGE-34773317-1	Section 1	-1.07	1.45	-1.22	2.18	-1.05	1.43	-1.46	2.29
	Section 2	-3.95	4.14	0.51	5.75	-3.13	4.93	0.48	5.86
	Section 3	1.39	2.18	0.91	2.74	0.66	2.72	0.30	2.82

Table S3. Comparison of QM and MM molecular polarizability for the three ligands using the Drude polarizable FF. QM molecular polarizabilities are scaled by a factor of 0.85.

Ligand	Subsections	QM Molecular Polarizability (Å³)				MM Molecular Polarizability (Å³)			
		α_{xx}	α_{yy}	α_{zz}	α_{iso}	α_{xx}	α_{yy}	α_{zz}	α_{iso}
MAYBRIDGE-SEW03510-3	Section 1	11.06	10.07	5.38	8.84	10.79	10.09	7.06	9.31
CHEMBRIDGE-57665815-3	Section 1	10.11	8.87	9.32	9.43	10.43	7.83	9.44	9.24
	Section 2	15.68	12.47	0.54	12.90	15.85	11.95	10.04	12.62
	Section 3	10.08	10.42	8.37	9.62	10.02	11.06	7.42	9.50
CHEMBRIDGE-34773317-1	Section 1	11.51	11.45	6.22	9.72	11.27	11.30	6.68	9.75
	Section 2	15.42	12.53	9.39	12.45	15.89	11.83	8.26	11.99
	Section 3	21.32	21.33	11.29	17.98	21.79	21.51	10.49	17.93

Table S4. Comparison of QM and MM ligand–water interaction energies for MAYBRIDGE-SEW03510-3 using the CHARMM FF. Minimum-energy interaction distances (r_{min}) are reported in Å and interaction energies (E_{int}) in kcal mol⁻¹.

Interaction	r_{min} (QM)	E_{int} (QM)	r_{min} (MM)	E_{int} (MM)	Δr_{min}	ΔE_{int}
C2 OOP	2.37	2.54	3.00	3.21	0.63	0.67
C4 OOP	2.81	4.36	3.00	3.66	0.19	−0.70
H7 180°	2.18	−4.51	2.51	−6.95	0.33	−1.44
H12 180°	1.81	−12.08	1.88	−12.16	0.07	−0.08
H1 180°	1.95	−8.18	2.06	−8.88	0.11	−0.70
N2 OOP	2.20	2.76	2.17	3.45	-0.03	0.69
H5 180°	2.05	−4.98	2.30	−3.95	0.25	1.03
N4 OOP	2.31	0.61	2.32	−0.11	0.01	−0.72
O 180°	1.83	−7.03	1.85	−6.53	0.02	0.50
H3 180°	1.81	−12.28	1.84	−12.20	0.03	0.08
H6180°	2.00	−5.26	2.50	−4.08	0.5	1.18

Table S5. Ligand–water interaction energies for CHEMBRIDGE-57665815-3 using the CHARMM FF. The ligand was divided into three subsections during parameter optimization. Minimum-energy interaction distances (r_{min}) are reported in Å and interaction energies (E_{int}) in kcal mol⁻¹.

Section	Interaction	r_{min} (QM)	E_{int} (QM)	r_{min} (MM)	E_{int} (MM)	Δr_{min}	ΔE_{int}
1	H6 180°	1.80	-14.88	1.77	-15.47	-0.03	-0.59
	H7 180°	1.80	-14.87	1.77	-15.47	-0.03	-0.60
	H15 180°	2.20	-5.30	2.53	-6.70	0.33	-1.40
	H17 180°	2.20	-5.29	2.53	-6.65	0.33	-1.36
	H3 180°	2.30	-3.80	2.63	-4.43	0.33	-0.63
	H9 180°	2.54	-9.80	3.00	-9.41	0.46	0.39
	H4 180°	2.33	-2.98	2.67	-3.13	0.34	-0.15
	H8 180°	2.47	-3.78	2.71	-4.49	0.24	-0.71
	H13 180°	2.33	-3.17	2.67	-3.33	0.34	-0.16
2	H1 180°	2.24	-4.22	2.58	-4.65	0.34	-0.43
	H13 180°	2.27	-4.72	2.59	-4.95	0.32	-0.23
	H3 180°	1.82	-12.11	1.88	-11.74	0.06	0.37
	C6 OOP	2.72	5.38	3.00	4.76	0.28	-0.62
	H26 180°	1.84	-10.80	1.90	-11.03	0.06	-0.23
	C1 OOP	2.63	2.23	3.00	1.67	0.37	-0.56
	H4 180°	1.95	-7.21	2.03	-8.21	0.08	-1.00
	H22 180°	2.23	-3.87	2.65	-3.39	0.42	0.48
	H23 180°	2.32	-5.79	2.72	-4.76	0.4	1.03
	H24 180°	2.34	-3.36	2.70	-3.39	0.36	-0.03
	H27 180°	2.30	-3.12	2.66	-2.95	0.36	0.17
3	H2 180°	1.85	-1.61	1.97	-2.70	0.12	-1.09
	C10 OOP	2.97	0.40	3.00	-2.07	0.03	-2.47
	C7 OOP	3.72	0.34	3.00	-2.01	-0.72	-2.35
	H1 180°	1.86	-2.13	2.01	-3.48	0.15	-1.35
	H20 180°	2.22	-0.23	2.69	-1.40	0.47	-1.17
	H24 180°	2.27	0.38	2.75	-0.69	0.48	-1.07

Table S6. Ligand–water interaction energies for CHEMBRIDGE-34773317-1 using the CHARMM FF. Minimum-energy interaction distances (r_{min}) are reported in Å and interaction energies (E_{int}) in kcal mol⁻¹.

Section	Interaction	r_{min} (QM)	E_{int} (QM)	r_{min} (MM)	E_{int} (MM)	Dr_{min}	ΔE_{int}
1	H18 180°	1.88	-1.78	2.00	-2.87	0.12	-1.09
	H19 180°	1.89	-1.59	2.01	-2.73	0.12	-1.14
	H2 180°	2.17	1.15	2.72	-0.19	0.55	-1.34
	H10 180°	2.18	0.68	2.78	0.02	0.6	-0.66
	H7 180°	2.18	0.68	2.78	0.02	0.6	-0.66
3	H32 180°	2.18	0.31	2.90	0.16	0.72	-0.15
	C2 OOP	2.16	-0.50	2.40	-1.52	0.24	-1.02
	H1 180°	2.06	-0.41	2.73	-1.11	0.67	-0.70
	C3 OOP	2.43	1.62	2.64	-0.24	0.21	-1.86
	H17 180°	1.72	-3.61	2.19	-3.20	0.47	0.41
	N2 OOP	2.03	-2.17	2.16	-3.11	0.13	-0.94
	C14 OOP	2.26	0.56	2.46	-0.44	0.2	-1.00
	H9 180°	2.04	-2.70	2.60	-2.91	0.56	-0.21
	H21 180°	1.69	-6.38	1.88	-7.42	0.19	-1.04

Table S7. Ligand–water interaction energies for MAYBRIDGE-SEW03510-3 using the Drude FF. Minimum-energy interaction distances (r_{min}) are reported in Å and interaction energies (E_{int}) in kcal mol⁻¹.

Interaction	r_{min} (QM)	E_{int} (QM)	r_{min} (MM)	E_{int} (MM)	Dr_{min}	ΔE_{int}
C2 OOP	2.37	-1.08	2.36	-0.58	-0.01	0.50
C4 OOP	2.81	2.24	3.00	2.77	0.19	0.53
H7 180°	2.18	-5.81	2.63	-5.41	0.45	0.40
H12 180°	1.81	-13.74	1.77	-12.82	-0.04	0.92
H1 180°	1.95	-9.45	1.91	-9.71	-0.04	-0.26
N2 OOP	2.20	-0.28	2.05	-1.10	-0.15	-0.82
H4 180°	2.45	-3.41	2.72	-4.28	0.27	-0.87
N4 OOP	2.31	-0.77	2.21	-0.82	-0.1	-0.05
O 180°	1.83	-8.55	1.84	-7.74	0.01	0.81
H3 180°	1.81	-13.83	1.73	-13.12	-0.08	0.71
N3 OOP	2.61	-0.18	2.49	-1.01	-0.12	-0.83
H6 180°	2.00	-7.12	2.01	-6.54	0.01	0.58

Table S8. Ligand–water interaction energies for CHEMBRIDGE-57665815-3 using the Drude FF. The ligand was divided into three subsections during parameter optimization. Minimum-energy interaction distances (r_{min}) are reported in Å and interaction energies (E_{int}) in kcal mol⁻¹.

Section	Interaction	r_{min} (QM)	E_{int} (QM)	r_{min} (MM)	E_{int} (MM)	Dr_{min}	ΔE_{int}
1	H6 180°	1.80	-16.12	1.58	-15.75	-0.22	0.37
	H7 180°	1.80	-16.11	1.58	-15.72	-0.22	0.39
	H14 180°	2.31	-6.24	2.59	-5.09	0.28	1.15
	H15 180°	2.20	-5.96	2.54	-5.35	0.34	0.62
	H16 180°	2.33	-6.48	2.59	-5.18	0.26	1.30
	H17 180°	2.20	-5.96	2.54	-5.29	0.34	0.67
	H3 180°	2.30	-4.58	2.63	-4.80	0.33	-0.22
	H9 180°	2.54	-11.56	2.85	-10.65	0.31	0.91
	H4 180°	2.33	-3.65	2.64	-3.81	0.31	-0.17
	H8 180°	2.47	-4.82	2.67	-3.95	0.2	0.87
	H13 180°	2.33	-3.93	2.65	-3.53	0.32	0.40
2	H1 180°	2.24	-4.22	2.61	-4.60	0.37	-0.38
	H2 180°	2.32	-5.45	2.75	-4.34	0.43	1.11
	H13 180°	2.27	-5.42	2.65	-4.42	0.38	1.00
	H3 180°	1.82	-13.66	1.73	-13.19	-0.09	0.47
	C6 OOP	2.72	1.90	3.00	1.56	0.28	-0.33
	H26 180°	1.84	-12.89	1.76	-12.42	-0.08	0.47
	C1 OOP	2.63	-1.14	2.67	-1.62	0.04	-0.48
	H4 180°	1.95	-8.83	1.92	-8.87	-0.03	-0.04
	H22 180°	2.23	-4.68	2.63	-3.79	0.4	0.89
	H23 180°	2.32	-7.08	2.63	-6.04	0.31	1.04
	H24 180°	2.34	-4.06	2.71	-3.50	0.37	0.56
	H27 180°	2.30	-3.81	2.67	-3.42	0.37	0.39
3	H2 180°	2.05	-3.01	2.05	-3.09	0	-0.08
	C10 OOP	3.17	-1.50	2.94	-2.36	-0.23	-0.86
	H1 180°	2.06	-3.49	2.02	-3.13	-0.04	0.36
	H20 180°	2.42	-1.25	2.75	-0.94	0.33	0.31
	H24 180°	2.47	-0.59	2.84	-0.46	0.37	0.13

Table S9. Ligand–water interaction energies for CHEMBRIDGE-34773317-1 using the Drude FF. The ligand was divided into three subsections during electrostatic parameter optimization. Minimum-energy interaction distances (r_{min}) are reported in Å and interaction energies (E_{int}) in kcal mol⁻¹.

Section	Interaction	r_{min} (QM)	E_{int} (QM)	r_{min} (MM)	E_{int} (MM)	Dr_{min}	ΔE_{int}
1	H18 180°	2.08	-2.90	2.11	-2.52	0.03	0.38
	H19 180°	2.09	-2.72	2.12	-2.40	0.03	0.32
	C6 OOP	2.44	-3.30	2.34	-3.70	-0.1	-0.40
	C4 OOP	2.35	-3.22	2.34	-2.45	-0.01	0.77
	H2 180°	2.38	-0.32	2.93	0.23	0.55	0.55
	H10 180°	2.38	-0.56	2.78	-0.49	0.4	0.07
	H7 180°	2.38	-0.56	2.78	-0.49	0.4	0.07
2	O1 180°	1.97	-5.44	1.93	-5.74	-0.04	-0.30
	C5 OOP	2.65	-0.79	2.47	-0.71	-0.18	0.08
	N5 OOP	2.27	-1.77	2.13	-1.76	-0.14	0.02
	H20 180°	2.02	-4.96	2.02	-4.28	0	0.68
	S 180°	2.82	-1.11	2.00	-0.41	-0.82	0.70
	H13 180°	2.25	-0.86	2.66	-0.58	0.41	0.28
	H15 180°	2.31	-1.48	2.70	-1.40	0.39	0.08
	H26 180°	2.36	-1.16	2.76	-0.97	0.4	0.19
	H29 180°	2.35	-0.57	2.68	-1.05	0.33	-0.48
	H30 180°	2.36	-1.57	2.68	-1.73	0.32	-0.16
	H32 180°	2.38	-0.71	2.82	-0.19	0.44	0.52
	H33 180°	2.37	-0.07	2.81	-0.35	0.44	-0.28
3	H34 180°	2.40	-0.83	2.77	-0.74	0.37	0.09
	C1 OOP	2.39	-3.52	2.42	-2.73	0.03	0.79
	C2 OOP	2.36	-3.53	2.39	-2.78	0.03	0.75
	H1 180°	2.26	-1.60	2.63	-0.94	0.37	0.66
	C3 OOP	2.63	-1.86	2.76	-1.34	0.13	0.52
	N1 OOP	2.38	-2.21	2.87	-1.42	0.49	0.79
	H17 180°	1.92	-5.38	1.95	-4.77	0.03	0.61
	N2 OOP	2.23	-3.99	2.13	-3.48	-0.1	0.51

	C8 OOP	2.54	-2.44	2.50	-2.10	-0.04	0.34
	C9 OOP	2.41	-2.98	2.30	-2.59	-0.11	0.39
	H4 180°	2.28	-1.60	2.55	-1.45	0.27	0.15
	C13 OOP	2.47	-2.54	2.39	-2.24	-0.08	0.30
	H8 180°	2.26	-1.43	2.52	-0.90	0.26	0.53
	C11 OOP	2.47	-2.61	2.49	-2.01	0.02	0.60
	C14 OOP	2.46	-2.14	2.44	-1.17	-0.02	0.97
	H9 180°	2.25	-2.95	2.43	-2.75	0.18	0.20
	C10 OOP	2.39	-3.01	2.37	-2.15	-0.02	0.86
	H5 180°	2.26	-2.17	2.44	-2.32	0.18	-0.15
	H21 180°	1.89	-6.79	1.94	-6.45	0.05	0.34

Table S10. Average binding site volumes (Å^3) of NtMGAM, CtMGAM, and α-amylase sampled over three replicate simulations using the CHARMM and Drude FFs.

Enzyme	Simulations	CHARMM	Drude
NtMGAM	Replicate 1	666 ± 68	499 ± 53
	Replicate 2	515 ± 84	582 ± 88
	Replicate 3	730 ± 65	633 ± 67
CtMGAM	Replicate 1	1205 ± 101	1197 ± 115
	Replicate 2	1135 ± 145	1105 ± 131
	Replicate 3	1140 ± 136	1472 ± 135
α-Amylase	Replicate 1	846 ± 84	473 ± 68
	Replicate 2	779 ± 77	655 ± 79
	Replicate 3	426 ± 83	606 ± 64
